# VqMAPK3/VqMAPK6, VqWRKY33, and *VqNSTS3* constitute a regulatory node in enhancing resistance to powdery mildew in grapevine

**DOI:** 10.1093/hr/uhad116

**Published:** 2023-05-31

**Authors:** Wandi Liu, Chaohui Yan, Ruimin Li, Guanyu Chen, Xinqi Wang, Yingqiang Wen, Chaohong Zhang, Xiping Wang, Yan Xu, Yuejin Wang

**Affiliations:** College of Horticulture, Northwest A & F University, Yangling, Shaanxi, 712100, China; Key Laboratory of Horticultural Plant Biology and Germplasm Innovation in Northwest China, Ministry of Agriculture, Yangling, Shaanxi, 712100, China; State Key Laboratory of Crop Stress Biology in Arid Areas, Northwest A & F University, Yangling, Shaanxi, 712100, China; College of Horticulture, Northwest A & F University, Yangling, Shaanxi, 712100, China; Key Laboratory of Horticultural Plant Biology and Germplasm Innovation in Northwest China, Ministry of Agriculture, Yangling, Shaanxi, 712100, China; State Key Laboratory of Crop Stress Biology in Arid Areas, Northwest A & F University, Yangling, Shaanxi, 712100, China; College of Horticulture, Northwest A & F University, Yangling, Shaanxi, 712100, China; Key Laboratory of Horticultural Plant Biology and Germplasm Innovation in Northwest China, Ministry of Agriculture, Yangling, Shaanxi, 712100, China; State Key Laboratory of Crop Stress Biology in Arid Areas, Northwest A & F University, Yangling, Shaanxi, 712100, China; College of Horticulture, Northwest A & F University, Yangling, Shaanxi, 712100, China; Key Laboratory of Horticultural Plant Biology and Germplasm Innovation in Northwest China, Ministry of Agriculture, Yangling, Shaanxi, 712100, China; State Key Laboratory of Crop Stress Biology in Arid Areas, Northwest A & F University, Yangling, Shaanxi, 712100, China; College of Horticulture, Northwest A & F University, Yangling, Shaanxi, 712100, China; Key Laboratory of Horticultural Plant Biology and Germplasm Innovation in Northwest China, Ministry of Agriculture, Yangling, Shaanxi, 712100, China; State Key Laboratory of Crop Stress Biology in Arid Areas, Northwest A & F University, Yangling, Shaanxi, 712100, China; College of Horticulture, Northwest A & F University, Yangling, Shaanxi, 712100, China; Key Laboratory of Horticultural Plant Biology and Germplasm Innovation in Northwest China, Ministry of Agriculture, Yangling, Shaanxi, 712100, China; State Key Laboratory of Crop Stress Biology in Arid Areas, Northwest A & F University, Yangling, Shaanxi, 712100, China; College of Horticulture, Northwest A & F University, Yangling, Shaanxi, 712100, China; Key Laboratory of Horticultural Plant Biology and Germplasm Innovation in Northwest China, Ministry of Agriculture, Yangling, Shaanxi, 712100, China; State Key Laboratory of Crop Stress Biology in Arid Areas, Northwest A & F University, Yangling, Shaanxi, 712100, China; College of Horticulture, Northwest A & F University, Yangling, Shaanxi, 712100, China; Key Laboratory of Horticultural Plant Biology and Germplasm Innovation in Northwest China, Ministry of Agriculture, Yangling, Shaanxi, 712100, China; State Key Laboratory of Crop Stress Biology in Arid Areas, Northwest A & F University, Yangling, Shaanxi, 712100, China; College of Horticulture, Northwest A & F University, Yangling, Shaanxi, 712100, China; Key Laboratory of Horticultural Plant Biology and Germplasm Innovation in Northwest China, Ministry of Agriculture, Yangling, Shaanxi, 712100, China; State Key Laboratory of Crop Stress Biology in Arid Areas, Northwest A & F University, Yangling, Shaanxi, 712100, China; College of Horticulture, Northwest A & F University, Yangling, Shaanxi, 712100, China; Key Laboratory of Horticultural Plant Biology and Germplasm Innovation in Northwest China, Ministry of Agriculture, Yangling, Shaanxi, 712100, China; State Key Laboratory of Crop Stress Biology in Arid Areas, Northwest A & F University, Yangling, Shaanxi, 712100, China

## Abstract

Grapevine powdery mildew is caused by *Erysiphe necator*, which seriously harms grape production in the world. Stilbene synthase makes phytoalexins that contribute to the resistance of grapevine against powdery mildew. A novel *VqNSTS3* was identified and cloned from Chinese wild *Vitis quinquangularis* accession Danfeng-2. The novel *VqNSTS3* was transferred into susceptible ‘Thompson Seedless’ by *Agrobacterium*-mediated transformation. The transgenic plants showed resistance to the disease and activated other resistance-related genes. *VqNSTS3* expression in grapevine is regulated by VqWRKY33, and which binds to TTGACC in the *VqNSTS3* promoter. Furthermore, VqWRKY33 was phosphorylated by VqMAPK3/VqMAPK6 and thus led to enhanced signal transduction and increased *VqNSTS3* expression. *ProVqNSTS3::VqNSTS3*-GFP of transgenic *VqNSTS3* in *Arabidopsis thaliana* was observed to move to and wrap the pathogen’s haustoria and block invasion by *Golovinomyces cichoracearum*. These results demonstrate that stilbene accumulation of novel *VqNSTS3* of the Chinese wild *Vitis quinquangularis* accession Danfeng-2 prevented pathogen invasion and enhanced resistance to powdery mildew. Therefore, *VqNSTS3* can be used in generating powdery mildew-resistant grapevines.

## Introduction

The grapevine is one of the ancient and most economically valuable fruits in the world [1]. Because of their high-quality fruit, European grape varieties (*Vitis vinifera*) are considered the world’s most prominent cultivars. However, this valuable species is highly susceptible to *Erysiphe necator* (previously *Uncinula necator*), a fungus that causes powdery mildew (PM) disease [2]. The obligate biotrophic fungus *E. necator* affects all parts of a plant, which leads to significant losses in fruit yield and quality in the viticulture industry [3–5]. The main measure for preventing and controlling grapevine PM in grape production is the spraying of chemical fungicides, which not only causes cost increases, fruit contamination, and environmental pollution, but also causes resistance in pathogenic bacteria and new variations in pathogenic bacteria, bringing new control difficulties to grape production [6–8]. Therefore, using the grapevine immune system to improve disease resistance is a fundamental way to solve the disease resistance problem in grape production. Obtaining PM-resistant grape varieties and elucidating the molecular mechanisms of disease resistance are vital steps in reducing the reliance on fungicides and breeding grapevine varieties for disease resistance.

An early study that first isolated resveratrol in grapevine leaves found that it had the effect of conferring disease resistance to grapevine [9]. *Vitis vinifera* was found to produce a stilbene phytoalexin, a derivative of resveratrol [10]. In later studies, resveratrol was isolated and obtained in grape berries [11] and wine [12]. There has been significant research on the role resveratrol plays in grapevine against *Botrytis cinerea* [13, 14], *E. necator* [15], *Plasmopara viticola* [16], and *Neofusicoccum parvum* [17]. Resveratrol also has benefit associated with human health [18, 19]. Stilbene synthase (STS) catalyzes the formation of resveratrol from three malonyl coenzyme A esters and one coenzyme A ester [20]. Heterologous expression of *STS* genes can improve the level of stilbene and enhance plant disease resistance. For example, transferring two *STS* genes from grapevine, where they are highly expressed, into tobacco plants, increased their resistance to *B. cinerea* [21]. Grapevine *STS* genes have been transferred to many plants, including rice [22], pea [23], lettuce [24], and kiwifruit [25], in each case resulting in significant improvements in accumulation of stilbene or pathogen resistance.

In recent years, several transcription factors were shown to regulate the expression of *STS* genes and stilbene accumulation, such as MYB, WRKY, ERF, AL, and bZIP [26–35]. Among them, WRKY transcription factors are important in regulating *STS* gene expression. VvWRKY24 can independently regulate the *VvSTS29* promoter, while VvWRKY03 and VvMYB14 jointly upregulate the *VvSTS29* promoter [35]. Recently, VvWRKY8 has been shown to repress *VvSTS15/21* expression and resveratrol biosynthesis through interaction with VvMYB14 [31]. When grapes are under UV stress, they produce resveratrol through VvMYB14-VvWRKY8-VvMYB30, and prevent excessive accumulation of resveratrol [29]. VqWRKY53 positively regulates the expression of *VqSTS*s, and interacts with VqMYB14 and VqMYB15 to show stronger regulatory function [32]. VqWRKY31 can be induced after *E. necator* and directly regulates the promoters of *STS9/48* [26]. However, it is currently unclear whether other WRKY transcription factors are involved in regulating *STS* gene expression.

In order to better study the traits and functions of grapevine genes, functional genome sequencing was conducted in different germplasms of grapes. In 2007, genome sequencing of PN40024 ‘Pinot Noir’ identified 48 *VvSTS* genes [1], of which 33 had potential functions [1, 36, 37]. Girollet *et al*. reported the *de novo* assembly of the *Vitis riparia* genome in 2019 [38], while an analysis of grapevine diversity and demographic history was performed using whole-genome resequencing of 472 *Vitis* accessions by Liang *et al*. [39]. The draft genome of *V. riparia* ‘Manitoba 37’, a native American cold-hardy grapevine, has been sequenced [40]. The genome of the grape interspecific hybrid ‘Shine Muscat’ (*Vitis labruscana* × *V. vinifera*) was sequenced and published in 2022 [41].

China is one of the main points of origin for grape varieties and has abundant germplasm resources that can be used for grape breeding [42]. Preliminary research in our laboratory found that Chinese wild *Vitis pseudoreticulata* accession Baihe-35-1 can provide a genetic resource to investigate the role of stilbene synthase genes in the PM interaction [43–45]. In total, 61 *VpSTS* genes have been isolated from Baihe-35-1 [46]. In particular, *VpSTS29/STS2* contributes to basal resistance of grapevine and *Arabidopsis thaliana* to PM [47, 48]. Another important Chinese wild resource is *Vitis quinquangularis* accession Danfeng-2, containing 41 *STS* genes (GenBank accession numbers JQ868658–JQ868698) [49]. Many *VqSTS* genes from Danfeng-2 have been shown to significantly enhance resistance to PM [50–53]. Among them, overexpression of the fruit-specific and highly expressed gene *VqSTS6* increases resveratrol content and pathogen resistance in *V. vinifera* ‘Thompson Seedless’ [53, 54]. Further analysis of Danfeng-2 novel transcriptome data (PRJNA306731) identified six novel *STS* transcripts: *VqNSTS1*–*VqNSTS6* [55]. VqAL4 positively regulates *VqNSTS4* expression, enhancing resistance to PM by activating salicylic acid (SA) signals in grapevine [34]. What is the mechanism of *VqNSTS3* expression in disease resistance? This research elucidated the effect and regulation mechanism of *VqNSTS3* in Chinese wild grape breeding for disease resistance.

## Results

### *VqNSTS3* has conserved motifs of the stilbene synthase gene family and expresses resistance to *E. necator*

**Figure 1:**
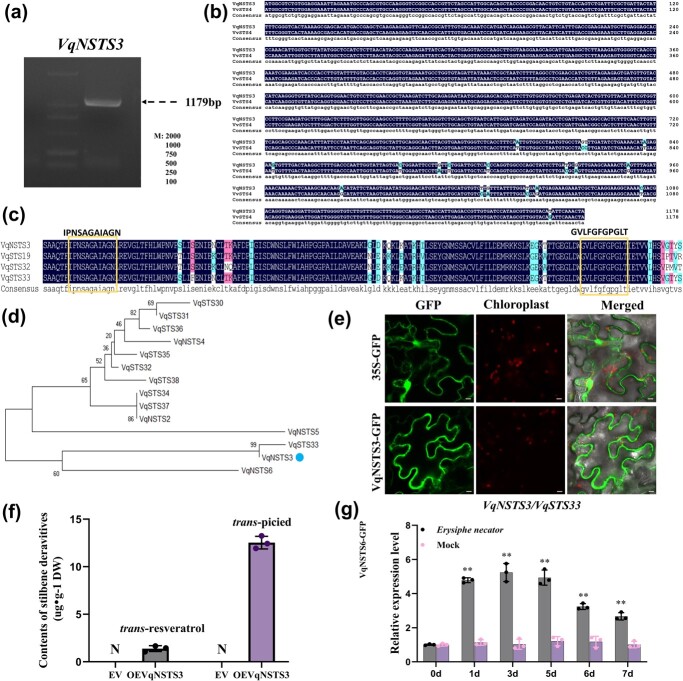
Cloning and expression analysis of *VqNSTS3* under *E. necator* inoculation in grapevine. **a** Amplification of *VqNSTS3* from Chinese wild *V. quinquangularis* accession Danfeng-2. **b** DNA sequence alignment between *VqNSTS3* and *VvSTS4* (XM_003634021). **c** Multiple amino acid sequence alignments between VqNSTS3 and other VqSTS proteins. The yellow box indicates the STS conserved domain. **d** Phylogenetic analysis of VqNSTS3 and part of VqSTSs from Danfeng-2. VqNSTS3 is highlighted with a blue dot. **e** Subcellular localization of VqNSTS3 in *N. benthamiana* leaves. Scale bars, 10 μm. **f***VqNSTS3* was transformed into tobacco for 3 days. The content of stilbenes was determined by HPLC. Results are shown as mean ± standard error of the mean; *n* = 3. **g** qPCR analysis of *VqNSTS3*/*VqSTS33* expression in Danfeng-2 leaves after infection with *E. necator*. Results are shown as mean ± standard error of the mean; *n* = 3. Significance was examined by one-way ANOVA followed by Dunnett’s multiple comparisons test (^**^P < .01).

Six new *STS* transcripts were identified in our laboratory [55]. A homologous cloning method was used to identify *VqNSTS3* (GenBank accession number OL589478) from Danfeng-2. The coding sequence of *VqNSTS3* was 1179 bp (Fig. 1a) and showed 98.3% similarity to *VvSTS4* from *V. vinifera* PN40024 (Fig. 1b). VqNSTS3 possessed the conserved domain of the STS family [56] (Fig. 1c) and showed 99% amino acid sequence identity with VqSTS33 [34] (Fig. 1d). VqNSTS3-GFP localized in the cytoplasm (Fig. 1e). Six new transcripts were transiently transformed into tobacco, in which we detected the highest content of stilbenes after overexpression of *VqNSTS3* (Fig. 1f) (data not shown). To further explore whether the gene responds to the induction of *E. necator* in Danfeng-2, samples from Danfeng-2 plants were taken for qPCR analysis after artificial inoculation with *E. necator*. It was found that *VqNSTS3* gene expression increased significantly on the first day after inoculation, and the trend continued to the third day after inoculation (Fig. 1g).

### Transgenic *VqNSTS3* grapevine lines show enhanced resistance to *E. necator* and activation of resistance-related genes

**Figure 2:**
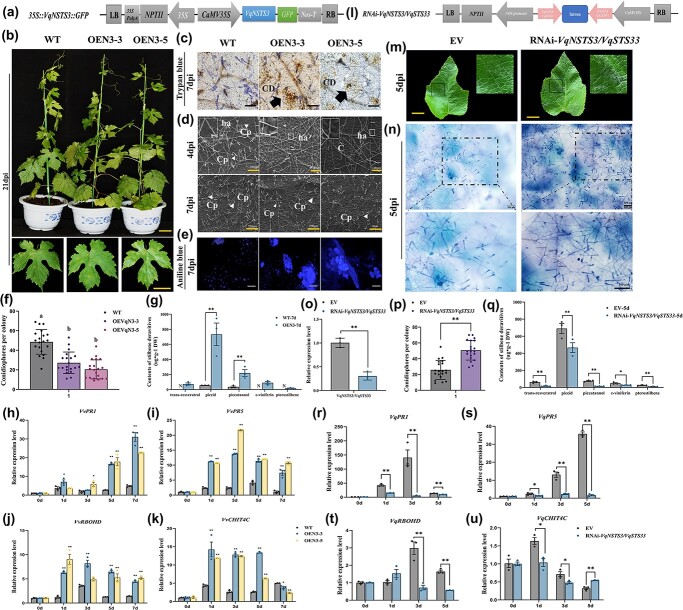
Transgenic *VqNSTS3* grapevine plants show enhanced resistance to *E. necator*. **a** Diagram of the OE*VqNSTS3* construct. **b** Photographs of *VqNSTS3* overexpression and WT plants infected with *E. necator* at 21 days post-inoculation (dpi)*.* Scale bars, 3 cm. **c** Trypan blue staining of OE*VqNSTS3* and WT leaves at 7 dpi to observe the growth of hyphae. Scale bars, 100 μm. CD, cell death. **d** Scanning electron micrographs of WT and OE*VqNSTS3* leaves at 4 and 7 dpi. C, conidium; ha, hyphal appressorium; Cp, conidiophore. **e** Aniline blue staining of WT and OE*VqNSTS3* leaves at 7 dpi to detect callose deposition. Scale bars, 50 μm. **f** Number of conidiophores per colony at 7 dpi on WT and transgenic plants. Results are shown as mean ± standard error of the mean; *n* = 20; different letters represent significant differences (*P* < .05) as determined by one-way ANOVA followed by Tukey’s multiple comparisons test. **g** HPLC analysis of content of stilbenes in WT and OE*VqNSTS3* leaves at 7 dpi. **h**–**k** Expression of defense-related genes determined by qPCR analysis in WT and transgenic plants after *E. necator* inoculation. Results are shown as mean ± standard error of the mean; *n* = 3. Significance was examined by one-way ANOVA followed by Dunnett’s multiple comparisons test (^*^P < .05; ^**^P < .01). **l** Diagram of the RNAi-*VqNSTS3/VqSTS33* construct. **m** Phenotypes of RNAi-*VqNSTS3/VqSTS33* and EV leaves infected with *E. necator* for 5 days. Scale bars, 3 cm. **n** RNAi-*VqNSTS3/VqSTS33* and EV leaves at 5 days stained with trypan blue. Scale bars, 50 μm. **o** qPCR analysis of *VqNSTS3*/*VqSTS33* expression in RNAi-*VqNSTS3/VqSTS33* and EV leaves. **p** Number of conidiophores per colony at 5 dpi on EV and RNAi leaves. Results are shown as mean ± standard error of the mean; *n* = 20. Significance was examined by Student’s *t*-test (^**^P < .01). **q** HPLC analysis of stilbenes in RNAi-*VqNSTS3/VqSTS33* and EV leaves at 5 dpi. **r*–*u** Expression of defense-related genes determined by qPCR analysis in EV and RNAi leaves after *E. necator* inoculation. Results in (g, o, q-u) are shown as mean ± standard error of the mean; *n* = 3. Significance was examined by Student’s *t*-test (^*^P < .05; ^**^P < .01).

To determine whether *VqNSTS3* is involved in grapevine resistance to *E. necator*, *VqNSTS3* from Danfeng-2 was transferred into disease-susceptible European grape cultivar ‘Thompson Seedless’ using *Agrobacterium tumefaciens*-mediated transformation (Fig. 2a, Supplementary Data Fig. S3). Two independent *VqNSTS3*-transgenic overexpression lines were obtained (OE*VqNSTS3*-L3 and OE*VqNSTS3*-L5) (Supplementary Data Fig. S3i and j). Transgenic and wild-type (WT) plants were inoculated with *E. necator* to characterize the disease resistance function of *VqNSTS3.* WT plants were more susceptible, producing extensive fungal hyphae and conidiophores, whereas transgenic lines were not (Fig. 2b–d and f). Furthermore, transgenic lines showed enhanced callose deposition (Fig. 2e) and increased the expression of resistance-related genes after inoculation (Fig. 2h–k). HPLC assays indicated that after inoculation only piceid and piceatannol were detected in WT plants, while five stilbenes accumulated in transgenic plants. The contents of piceid and piceatannol in *VqNSTS3*-transgenic overexpression lines increased 13.0- and 6.3-fold, respectively, compared with WT plants (Fig. 2g, Supplementary Data Table S7). To study the role of *VqNSTS3* in disease resistance further, we used RNA interference (RNAi) to study the resistance of *VqNSTS3* to *E. necator* in Danfeng-2. Due to high sequence similarity between *VqNSTS3* and *VqSTS33*, we were only able to interfere with both at the same time. RNAi-*VqNSTS3/VqSTS33* plants showed contrasting results to the overexpressing (OE) plants. Following inoculation, *trans*-resveratrol, piceid, pterostilbene, *ε*-viniferin, and piceatannol levels in RNAi-*VqNSTS3/VqSTS33* were 27, 42, 57, 59, and 14% lower than in empty vector (EV) controls (Fig. 2l–u; Supplementary Data Table S7). The above results show that *VqNSTS3* plays an active role in the defense response after inoculation with *E. necator*.

### *VqNSTS3* expression enhances resistance to *E. necator* in grapevine due to regulation by VqWRKY33

**Figure 3:**
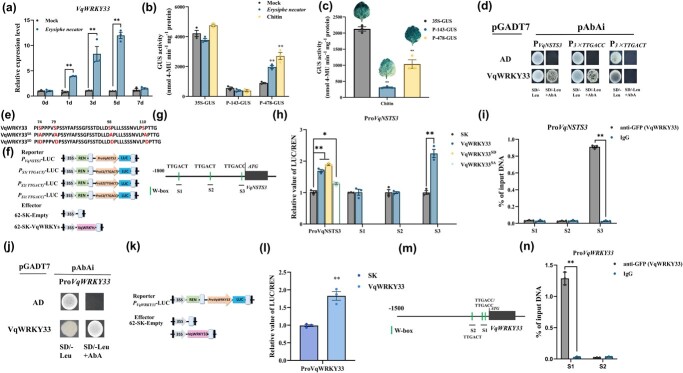
VqWRKY33 responds to *E. necator* and regulates *VqNSTS3* expression. **a** qPCR analysis of *VqWRKY33* expression in Danfeng-2 leaves after inoculation with *E. necator* for 0, 1, 3, 5, and 7 days. **b** Measurement of GUS activity. Danfeng-2 leaves expressing P-478-GUS and P-143-GUS were inoculated with *E. necator* or treated with chitin. **c** Chitin-induced GUS activity in transient expression tobacco leaves. Tobacco leaves expressing P-478-GUS and P-143-GUS were treated with 1 mg/ml chitin for 30 minutes. **d** Y1H analysis using pGADT7-VqWRKY33 as the prey and P*pVqNSTS3*-AbAi, P*3xTTGACT*-AbAi, and P*3xTTGACC*-AbAi as baits to demonstrate VqWRKY33 can bind to Pro*VqNSTS3* and TTGACC. **e** Loss-of-phosphorylation VqWRKY33 mutant with all four Ser residues mutated to Ala (VqWRKY33^SA^), and the phospho-mimicking VqWRKY33 mutant with all four Ser residues mutated to Asp (VqWRKY33^SD^). **f** Structural diagrams of dual-luciferase assays. **g** Schematic diagram of the promoter region of *VqNSTS3*. **h** Ratio of luciferase activity of VqWRKY33, VqWRKY33^SA^, and VqWRKY33^SD^ binding to the *VqNSTS3* promoter. **i** VqWRKY33 binding to the promoter of *VqNSTS3 in vivo* after *E. necator* treatment verified by ChIP–qPCR assays. **j** Y1H analysis using pGADT7-VqWRKY33 as prey and P*pVqWRKY33*-AbAi as bait to demonstrate VqWRKY33 can bind to its own promoter. **k** Structural diagrams of dual-luciferase assays. **l** Ratio of luciferase activity of VqWRKY33 binding to its own promoter. **m** Schematic diagram of the promoter region of *VqWRKY33*. **n** VqWRKY33 binding to promoter of the *VqWRKY33 in vivo* after *E. necator* inoculation, shown by ChIP–qPCR assays. Results in (a-c, h) are shown as mean ± standard error of the mean; *n* = 3. Significance was examined by one-way ANOVA followed by Dunnett’s multiple comparisons test (^*^P < .05; ^**^P < .01). Results in (i, l, n) are shown as values ± standard error of the mean; *n* = 3. Significance was examined by Student’s *t*-test (^**^P < .01).

We analyzed the *VqNSTS3* promoter cloned from Danfeng-2 to identify transcription factors that regulate *VqNSTS3* expression. The *VqNSTS3* promoter was found to contain three specific fungal elicitor-responsive elements: W-box elements (Supplementary Data Fig. S2, Supplementary Data Table S4), and the W-box element is the binding site of the WRKY transcription factor [57]. Previous studies in our laboratory found that 16 VqWRKY transcription factors responded to the induction of *E. necator* in Danfeng-2 [32]. To determine whether WRKY transcription factors could regulate the expression of *VqNSTS3*, we selected eight WRKY transcription factors that showed significant responses to the induction of *E. necator* using dual-luciferase assays to detect the promoter activity of *VqNSTS3*. The results show that VqWRKY2, VqWRKY18, VqWRKY33, and VqWRKY53 can positively activate the promoter activity of *VqNSTS3* and that VqWRKY33 had the greatest regulatory activity on the *VqNSTS3* promoter (Supplementary Data Fig. S5a and b). Therefore, we conducted further research on VqWRKY33. We artificially inoculated Danfeng-2 leaves with *E. necator*, and the expression of *VqWRKY33* was significantly upregulated after inoculation (Fig. 3a). To further investigate the function of the transcription factor VqWRKY33, the characteristics of *VqWRKY33* were analyzed. VqWRKY33 is a nuclear protein that encodes 561 amino acids and has two highly conserved WRKY domains (amino acid residues 230–285 and 392–449), predicted to be located on chromosome 8 (Supplementary Data Fig. S5c–f). The *VqWRKY33* promoter also contained three W-box elements located 143–478 bp upstream of the start codon (Supplementary Data Fig. S4, SupplementaryData Table S5). Two GUS fragments, P-478-GUS (with the W-box) and P-143-GUS (without the W-box), were constructed to determine whether the W-box of the *VqWRKY33* promoter could respond to chitin and *E. necator* (Supplementary Data Fig. S4a). Two GUS fragments were transiently transformed into the leaves of Danfeng-2 or tobacco, which were then artificially inoculated with *E. necator* or sprayed with chitin. The findings showed that the fragments containing W-box regions responded to *E. necator* and chitin (Fig. 3b and c). To test whether VqWRKY33 could bind to the *VqNSTS3* promoter, yeast one-hybrid (Y1H) assays were developed. It was found that VqWRKY33 can regulate the promoter of *VqNSTS3* by binding at TTGACC (Fig. 3d). Moreover, dual-luciferase assays were performed, which gave the same results (Fig. 3f–h). Phosphorylation sites in the SP cluster of VqWRKY33 are well known [58]. A mutation of the four Ser residues to Ala in VqWRKY33 blocked its binding to the *VqNSTS3* promoter, while a mutation of Ser to Asp enhanced its binding to the *VqNSTS3* promoter (Fig. 3e and h). Chromatin immunoprecipitation (ChIP)–qPCR was conducted to examine the binding of VqWRKY33 in the promoter of *VqNSTS3 in vivo* (Fig. 3g and i). Because of the presence of W-box elements in the *VqWRKY33* promoter, it was speculated that VqWRKY33 could regulate its own expression. Through Y1H assays, VqWRKY33 was found to regulate its own expression by binding to its own promoter (Fig. 3j). Moreover, the dual-luciferase and ChIP–qPCR assays obtained the same results (Fig. 3k–n). The above results show that VqWRKY33 positively regulates *VqNSTS3* expression by binding to TTGACC in the *VqNSTS3* promoter and also regulates its own expression.

### Transfer of *VqWRKY33* into ‘Thompson Seedless’ to promote resistance to *E. necator* through accumulation of stilbenes

**Figure 4:**
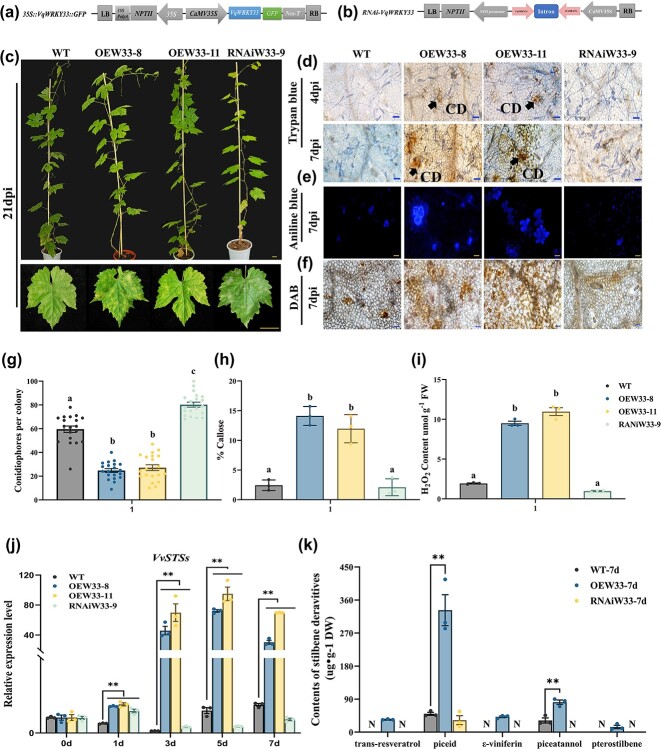
Overexpression of *VqWRKY33* in ‘Thompson Seedless’ promotes expression of *STS*s and resistance to *E. necator*. **a**, **b** Diagram of the (**a**) OE*VqWRKY33* and (**b**) RNAi*WRKY33* construct. **c** Photographs of OEW33-8, OEW33-11, RiW33-9, and WT plants at 21 dpi. Scale bars, 3 cm. **d** Trypan blue-stained OEW33-8, OEW33-11, RiW33-9, and WT leaves at 4 and 7 dpi. CD, cell death. Scale bars, 50 μm. **e** Aniline blue staining of OEW33-8, OEW33-11, RiW33-9, and WT leaves to detect callose deposition at 7 dpi. Scale bars, 50 μm. **f** DAB staining of OEW33-8, OEW33-11, RiW33-9, and WT leaves to detect H_2_O_2_ accumulation at 7 dpi. Scale bars, 50 μm. **g** Number of conidiophores per colony on OEW33-8, OEW33-11, RiW33-9, and WT leaves at 7 dpi. Results are shown as mean ± standard error of the mean; *n* = 20, and different letters represent significant differences (*P* < .05) as determined by one-way ANOVA followed by Tukey’s multiple comparisons test. **h** Quantification of callose deposition area on leaves at 7 dpi. **i** H_2_O_2_ content in OEW33-8, OEW33-11, RiW33-9, and WT leaves at 7 dpi. **j** qPCR analysis of *VvSTS* expression in OEW33-8, OEW33-11, RiW33-9, and WT plants after inoculation. Results are shown as mean ± standard error of the mean; *n* = 3. Significance was examined by one-way ANOVA followed by Dunnett’s multiple comparisons test (^**^P < .01). **k** HPLC analysis of stilbenes in (OE and RNAi) *WRKY33* and WT after inoculation. Results are shown as mean ± standard error of the mean; *n* = 3. Significance was examined by Student’s *t*-test (**P < .01). Results in (**h**) and (**i**) are shown as mean ± standard error of the mean; *n* = 3, and different letters represent significant differences (*P* < .05) as determined by one-way ANOVA followed by Tukey’s multiple comparisons test.

To determine the role of VqWRKY33 in the accumulation of stilbenes, two transgenic lines and one RNAi line were obtained by stable genetic transformation mediated by *A. tumefaciens* (Fig. 4a and b, Supplementary Data Fig. S6). Transgenic plants were identified by qPCR and western blot assays (Supplementary Data Fig. S6d and e), and the plants obtained were subjected to inoculation with *E. necato*r to observe the phenotypes; WT plants were used as negative control. After artificial inoculation, WT and RNAi plants showed more colonies than those of OE lines. In transgenic lines a more obvious hypersensitive response (HR) cell death phenotype could be observed (Fig. 4c, d, and g). OE plants accumulated more callose and H_2_O_2_ than in WT and RNAi plants by histochemical staining (Fig. 4e–f and h–i). The expression of *STS*s after inoculation in transgenic plants showed a more significant response than in the WT and RNAi plants (Fig. 4j). VqWRKY33’s influence on the accumulation of stilbenes after *E. necator* inoculation was further investigated. The HPLC assay indicated that after inoculation, only piceid and piceatannol were detected in WT plants, while five stilbenes accumulated in transgenic plants. The contents of piceid and piceatannol in *VqWRKY33* overexpression lines were 6.7 and 2.3 times higher, respectively, compared with WT. However, in RNAi-*WRKY33* we only detected piceid after inoculation (Fig. 4k, Supplementary Data Table S8). Overall, these results showed that VqWRKY33 is an important transcription factor in regulating the stilbene synthesis pathway.

### VqWRKY33 induces enhanced expression of *VqNSTS3* due to interaction with and phosphorylation by VqMAPK3/6

**Figure 5:**
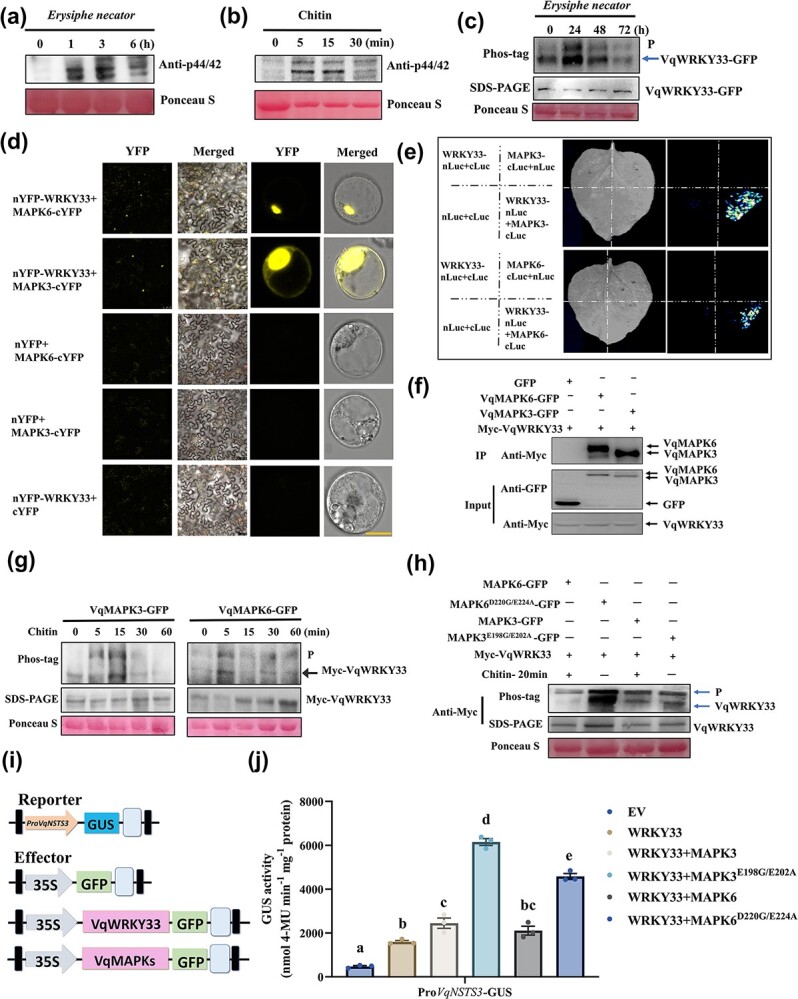
VqMAPK3/6 interact with and phosphorylate VqWRKY33, inducing the expression of *VqNSTS3*. **a** Activation of VqMAPKs in Danfeng-2 leaves treated with *E. necator* was verified by western blot assays. **b** Activation of VqMAPKs in Danfeng-2 leaves treated with 1 mg/ml chitin was verified by western blot assays. **c** Phosphorylation of VqWRKY33 was induced by *E. necator*. Proteins were separated by Phos-tag gel. **d** BiFC assays verified the interaction between VqWRKY33 and VqMAPK3/6 in tobacco leaves and grape protoplasts. Scale bars, 50/10 μm. **e** Split-luciferase complementation assays confirmed the interaction between VqWRKY33 and VqMAPK3/6. **f** CoIP assays validated that VqWRKY33 interacted with VqMAPK3 and VqMAPK6. **g** Phosphorylation of VqWRKY33 co-expressed with VqMAPK3 and VqMAPK6 after chitin treatment. Proteins were separated by Phos-tag gel, and then detected by immunoblotting with an anti-Myc antibody. **h** Phosphorylation of VqWRKY33 was induced by phospho-mimicking VqMAPK3/6 mutants. Proteins were separated by Phos-tag gel, and then detected by immunoblotting with an anti-Myc antibody. **i** Structural diagrams of GUS activity assays. **j** Measurement of GUS activity. Pro*VqNSTS3*-GUS was co-transformed with 35S-GFP, 35S-VqWRKY33-GFP, and 35S-VqMAPKs-GFP in tobacco leaves. Results are shown as mean ± standard error of the mean; *n* = 3, and different letters represent significant differences (*P* < .05) as determined by one-way ANOVA followed by Tukey’s multiple comparisons test.

Our previous study found that *MAPKKK38* responded significantly after *E. necator* induction [59]. We quantitatively analyzed *MAPKKK38* and five *MEKK* genes after inoculation with *E. necator* in Danfeng-2. Here, *MAPKKK38*, *MEKK3*, and *MEKK5* were significantly induced after *E. necator* inoculation (Supplementary Data Fig. S1). To further investigate VqWRKY33’s molecular role in regulating *VqNSTS3* in response to *E. necator*, we focused on protein–protein interaction networks. The STRING database was used to predict the possible interacting proteins of VqWRKY33, including MAPK3 and MAPK6 (Supplementary Data Fig. S7a–c, Supplementary Data Table S6). VqMAPK3, VqMAPK4, and VqMAPK6 could be activated by *E. necator* and chitin [60] (Fig. 5a and b); meanwhile, VqWRKY33 could be phosphorylated after inoculation with *E. necator* (Fig. 5c). Next, to determine whether VqWRKY33 could interact with VqMAPK3 and VqMAPK6, a BiFC assay was performed, allowing direct interaction between VqMAPK3/6 and VqWRKY33 to be observed in nuclei (Fig. 5d). A split-luciferase complementation assay and a co-immunoprecipitation (CoIP) assay also confirmed the interaction between VqMAPK3/6 and VqWRKY33 (Fig. 5e–f). To determine whether VqMAPK3 and VqMAPK6 could phosphorylate VqWRKY33, we separately co-expressed VqMAPK3 and VqMAPK6 with VqWRKY33 in tobacco leaves and sprayed them with chitin. Phos-tag gel was used to separate the extracted protein. Under chitin treatment, VqWRKY33 can be phosphorylated by VqMAPK3 and VqMAPK6 (Fig. 5g). Constitutively active MAPK3 and MAPK6 (MAPK3^CA^ and MAPK6^CA^), which result from two mutations (E198G/E202A and D220G/E224A) in each of the conserved domains, respectively, retain their substrate specificity and physiological functions [61] (Supplementary Data Fig. S7d and e). MAPK3/6^CA^ could phosphorylate VqWRKY33 without chitin treatment (Fig. 5h), which confirms the previous report that MAPK3/6^CA^ is a constitutively active form of MAPK3/6 [61]. To explore how VqMAPK3/6 affected VqWRKY33 function and regulated *VqNSTS3* expression, transient transactivation was performed via assays using the *VqNSTS3* promoter fused to GUS (Pro*VqNSTS3*-GUS). Co-expression of VqMPK3/6 induced VqWRKY33-activated *VqNSTS3* expression, and the VqMAPK3/6 constitutive activation form displayed enhanced *VqNSTS3* expression activity (Fig. 5i and j).

### VqMAPK3/6 positively regulate the expression of *VqSTS*s and enhance resistance to *E. necator* in grapevine

**Figure 6:**
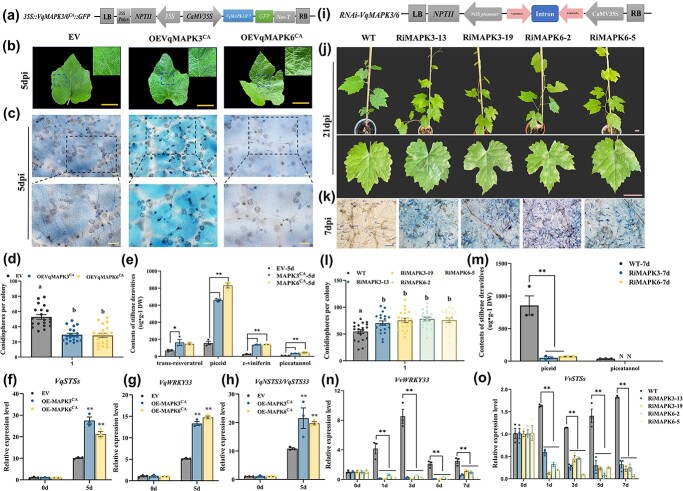
VqMAPK3/6 positively regulate the expression of *STS*s and enhance the disease resistance of grapevine. **a** Diagram of the OEV*qMAPK3/6^CA^* construct. **b** Photographs of OE*VqMAPK3/6^CA^* and EV leaves at 5 dpi. Scale bars, 3 cm. **c** Trypan blue-stained EV and OE*VqMAPK3/6^CA^* leaves at 5 dpi. Scale bars , 50 μm. **d** Number of conidiophores per colony on EV and OE*VqMAPK3/6^CA^* leaves at 5 dpi. **e** HPLC analysis of stilbenes in OE*VqMAPK3/6^CA^* and EV leaves. **f**–**h** qPCR analysis of *VqWRKY33*, *VqNSTS3/VqNSTS33*, and *VqSTS* in EV and OE leaves after *E. necator* inoculation. **i** Diagram of the RNAi-*MAPK3/6* construct. **j** Photographs of Ri*MAPK3-13*, Ri*MAPK3-19*, Ri*MAPK6-2*, Ri*MAPK6-5*, and WT plants at 21 dpi. Scale bars, 3 cm. **k** Trypan blue-stained RNAi-*MAPK3/6* and WT leaves at 7 dpi. Scale bars, 100 μm. **l** Number of conidiophores per colony on RNAi-*MAPK3/6* and WT leaves at 7 dpi. **m** HPLC analysis of stilbenes in RNAi-*MAPK3/6* and WT. **n**, **o** Expression of *VvSTS*s and *VvWRKY33* analyzed by qPCR in WT and RNAi-*MAPK3/6* plants after *E. necator* inoculation. In (**d**) and (**l**) results are shown as mean ± standard error of the mean; *n* = 20, and different letters represent significant differences (*P* < .05) as determined by one-way ANOVA followed by Tukey’s multiple comparisons test. In (**e**–**h**) and (**m**–**o**) results are shown as mean ± standard error of the mean; *n* = 3. Significance was examined by one-way ANOVA followed by Dunnett’s multiple comparisons test (^*^P < .05; ^**^, *P* < .01).

As VqMAPK3 and VqMAPK6 can be activated after *E. necator* inoculation (Fig. 5a), we speculated that VqMAPK3 and VqMAPK6 are involved in resistance to *E. necator*. 35S-VqMAPK3^CA^-GFP and 35S-VqMAPK6^CA^-GFP were transiently overexpressed in Danfeng-2 (EV as a negative control) (Fig. 6a, Supplementary Data S8c and d). We observed fewer spores on OE leaves than on the negative control after inoculation (Fig. 6b–d). The expression of *VqWRKY33*, *VqNSTS3/VqSTS33*, and *VqSTS*s in transient overexpression leaves was prominently higher than in the EV leaves (Fig. 6f–h). Contents of *trans*-resveratrol, piceid, *ε*-viniferin, and piceatannol in OE*VqMAPK3^CA^* leaves were 2.4-, 3.6-, 5.7-, and 1.3-fold compared with EV leaves after inoculation; in OE*VqMAPK6^CA^* leaves contents were 1.9-, 4.7-, 5.8-, and 1.6-fold compared with EV leaves after inoculation (Fig. 6e, Supplementary Data Table S9). These results indicated that phosphorylated VqMAPK3/6 could positively regulate the expression of *VqWRKY33* and *VqSTS*s and the production of stilbenes. *MAPK3* and *MAPK6* were then silenced in grapevines (Fig. 6i, Supplementary Data Fig. S8e and f). Notably, spores of *E. necator* on RNAi-*MAPK3* or RNAi-*MAPK6* leaves were strikingly larger than those on WT after inoculation (Fig. 6j–l). RNAi-*MAPK3* or RNAi-*MAPK6* significantly reduced the expression levels of *VvWRKY33* and *VvSTS*s (Fig. 6n and o). HPLC was performed to detect the accumulation of stilbenes in the RNAi lines, and only piceid was detected after inoculation. The contents of piceid in RNAi-*MAPK3/6* increased 4.2- and 11.9-fold, respectively, after inoculation. The results showed that the non-toxic piceid in susceptible plants was the main stilbene accumulated (Fig. 6m, Supplementary Data Table S9). Collectively, these results indicate that VqMAPK3/6 positively regulate *VqNSTS3* expression and increase the accumulation of stilbenes against *E. necator*.

### *Pro* *VqNSTS3::VqNSTS3*-GFP moves to and wraps the pathogen haustoria, forming encasements to block the invasion of pathogens in *A. thaliana*

**Figure 7:**
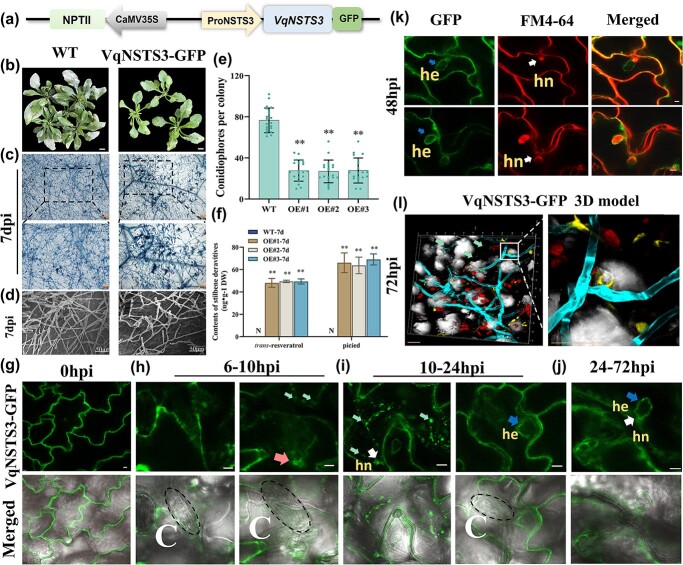
*ProVqNSTS3::VqNSTS3*-GFP moves to and wraps the pathogen haustoria to block the invasion of *G. cichoracearum* in *A. thaliana*. **a** Diagram of the *ProVqNSTS3::VqNSTS3*-GFP construct. **b** Photographs of *ProVqNSTS3::VqNSTS3*-GFP overexpression and Col-0 leaves infected with *G. cichoracearum* at 7 dpi. Scale bars, 1 cm. **c** Trypan blue staining of leaves from Col-0 and transgenic *A. thaliana* at 7 dpi. Scale bars, 100/50 μm. **d** Scanning electron micrographs of Col-0 and transgenic *A. thaliana* leaves inoculated with *G. cichoracearum* for 7 days. Scale bars, 50 μm. **e** Quantification of *G. cichoracearum* growth on *A. thaliana* leaves at 7 dpi. Results are shown as mean ± standard error of the mean; *n* = 20. Significance was examined by one-way ANOVA followed by Dunnett’s multiple comparisons test (^**^P < .01). **f** HPLC analysis of piceid and *trans*-resveratrol in *ProVqNSTS3::VqNSTS3*-GFP transgenic plants and Col-0. Results are shown as mean ± standard error of the mean; *n* = 3. Significance was examined by one-way ANOVA followed by Dunnett’s multiple comparisons test (^**^P < .01). **g**–**j** Confocal microscope images from single optical sections of *A. thaliana* leaf epidermal cells expressing *ProVqNSTS3::VqNSTS3*-GFP infected by *G. cichoracearum*. The top row shows *ProVqNSTS3::VqNSTS3*-GFP fluorescence and the bottom row shows the merged field images. The red arrow indicates the penetration site, green arrows indicate multivesicular bodies, white arrows indicate the haustorial necks, and blue arrows indicate haustorial encasements. hn, haustorial neck; he, haustorial encasement; C, conidium. Scale bars, 5 μm. **k***Arabidopsis thaliana* plants overexpressing *ProVqNSTS3::VqNSTS3*-GFP were inoculated with *G. cichoracearum* and haustorial encasements were analyzed by confocal microscopy at 48 hours post-inoculation (hpi) for PM. hn, haustorial neck; he, haustorial encasement. Scale bars, 5 μm. The left column shows *ProVqNSTS3::VqNSTS3*-GFP fluorescence, the middle column shows corresponding red fluorescence after staining with the membrane-specific tracer FM4-64, and the right column shows the corresponding merged images. **l** Z-projections through the epidermal cell layer were visualized by confocal microscopy at 72 hpi. They were 3D-reconstructed and displayed as maximum intensity projections. Image shows overlays of GFP fluorescence (yellow) and calcofluor white staining (cyan). Scale bars, 50 μm.

As the model plant *A. thaliana* does not contain *STS* genes, *VqNSTS3* was linked to its own promoter to stably transform *A. thaliana*, thus revealing the expression and function of the *VqNSTS3* gene in transgenic *A. thaliana* (Fig. 7a,  Supplementary Data Fig. S9). Transgenic lines of the *T*_3_ generation were artificially inoculated with *G. cichoracearum*, and after trypan blue staining it was found that large areas of HR cell death appeared on transgenic *A. thaliana* after inoculation (Fig. 7b and c). Furthermore, the number of spores on the transgenic lines was less than on WT (Fig. 7d and e). *Trans*-resveratrol and piceid in transgenic lines accumulated after inoculation (Fig. 7f, Supplementary Data Table S10). Preliminary studies have shown that stilbenes can inhibit the growth of hyphae, and mainly accumulate at the place where *G. cichoracearum* invades [15]. However, there is no direct evidence of how the *STS* gene resists *G. cichoracearum* infection. Forty-eight hours after inoculation of the OE lines, green fluorescence of VqNSTS3 was observed on the plasma membrane and the intact haustorium encasement. The lipophilic dye FM4-64 mainly marked the cell phospholipid membrane. Green and red fluorescence had obvious fluorescence overlap on the haustorial neck and plasma membrane (Fig. 7k). The yellow fluorescence was observed gathered around the secondary haustorium developing from new appressoria along the hyphae 72 hours after inoculation with *G. cichoracearum* (Fig. 7l). To further determine when and how *ProVqNSTS3::VqNSTS3*-GFP accumulated at haustorium encasements, a time-process study was conducted. At the invasion site of *G. cichoracearum*, aggregation of GFP fluorescence could be seen 6–10 hours post-inoculation (hpi), accompanied by the germination of spores. With increasing invasion time, some small multivesicular body (MVB) structures aggregated in the infected sites and started accumulating at the haustorial neck (10–24 hpi). A cupular encasement then formed around the haustorium [62]. Finally, the haustorium was completely wrapped by *ProVqNSTS3::VqNSTS3*-GFP (24–72 hpi) (Fig. 7g–j). These results indicate that stilbene synthase directly interacts with spores and inhibits the germination and growth of spores.

## Discussion

### On the stilbene synthase genes and novel *VqNSTS3* resistance to disease in grapevine

STS is a key enzyme in the biosynthesis of resveratrol [63]. Resveratrol in grapevine plays a key role in plant disease resistance and is beneficial to human health [18, 19]. After infection or stress in grapevine, resveratrol, a major stilbene product, accumulates in large amounts in the stressed areas [64]. We recently found six novel transcripts of *STS*s by analyzing the transcriptome data of Danfeng-2 [55]. Based on sequence alignment, there are significant differences between *VqNSTS2–6* and the reported *VqSTS*s, indicating that the *VqNSTS* genes are new members in Danfeng-2 [34]. Among them, *VqNSTS3* produced the highest stilbene content after it was transiently transformed into tobacco (Fig. 1f). Therefore, we speculated that *VqNSTS3* enhanced the resistance of Danfeng-2 to *E. necator* by accumulating stilbenes. In order to better study the function of *VqNSTS3* from Chinese wild grapevine in European grapevine varieties, an *A. tumefaciens*-mediated genetic regeneration system for transgenic grapevines was used for the identification of gene function in transgenic lines [43, 65]. We genetically transformed *VqNSTS3* into *V. vinifera* ‘Thompson Seedless’ and detected the accumulation of stilbenes after inoculation. The content of these chemicals in the *VqNSTS3* transgenic lines exceeded that in WT plants (Fig. 2g). Similar results were observed in *VpSTS29/STS2* [47] and *VqNSTS4* overexpression lines [34]. Plants resist pathogens through two layers of innate immunity: PAMP-triggered immunity (PTI) and effector-triggered immunity (ETI) [66–68]. PTI is the basic defense of plants, characterized by activation of multiple immune responses [69–73]. Callose deposits, a sign of the plant PTI response [74], are accumulated at the sites of attack during early stages of pathogen invasion [75]. The HR may inhibit or delay further spread of the pathogen [76]. Compared with WT plants, *VqNSTS3* overexpression plants exhibited more cell death and higher callose accumulation after *E. necator* infection (Fig. 2c–e). Consistent with this, *VqNSTS3*-transgenic *A. thaliana* plants showed HR cell death and limited spore growth and germination (Fig. 7b–e). These results were also found in previous studies [34, 47, 53]. SA signaling is another vital signal for plant immunity [77]. Overexpression of *VqNSTS3* in transgenic grapevine lines activated SA-related signaling genes *PR1* and *PR5* (Fig. 2h and i) and disease resistance-related genes *RBOHD* and *CHIT4C* (Fig. 2j and k), which is similar to the findings in *VqNSTS4* overexpression grapevines in that the plants showed enhanced disease resistance-related gene expression and enhanced resistance to *E. necator* [34]. In RNAi-*VqNSTS3* plants, however, we observed the opposite results (Fig. 2l–u). Overall, these results suggest that overexpression of *VqNSTS3* triggered several mechanisms after elicitor perception and regulated stilbene production in plant cells to enhance *E. necator* resistance [47, 78, 79]. Our results demonstrated that *VqNSTS3 *transgenic overexpression plants showed resistance-related gene expression, HR cell death, and callose deposition after inoculation by *E. necator*.

### Transcription factor regulation and novel mechanism of VqWRKY33 in grapevine

Several transcription factors involved in the regulation of grape *STS* genes have been discovered, including MYB, WRKY, ERF, and bZIP [26–33, 80]. Höll *et al.* reported that the MYB transcription factors that regulate the *STS* genes in grapevine via a typical path, VvMYB14 and VvMYB15, can activate the promoters of *VvSTS29/41* [30]. Jiang *et al*. revealed that VqMYB154 can promote polygene *VqSTS9/32/42* expression by binding to their promoters [27]. Aside from MYB transcription factors, WRKY transcription factors are also vital regulators of *STS* genes [35, 81]. VvWRKY24 alone can activate the promoter of *VvSTS29*, but VvWRKY3 needs to form an integrated organization with VvMYB14 to regulate *VvSTS29* [35]. *VqSTS32*/*41* are positively regulated by VqWRKY53; meanwhile VqWRKY53 interacts with VqMYB14 and VqMYB15 to show a stronger regulatory function [32]. VqWRKY31 can directly bind to the promoters of *STS9*/*48* [26]. The WRKY transcription factors are prominent signaling proteins participating in resistance to various fungal diseases in plants [82–85]. They are key regulatory components of plant disease resistance for *A. thaliana* [86], rice (*Oryza sativa*) [87], apple (*Malus domestica*) [85], *Brassica napus* [88], and rose (*Rosa hybrida*) [89]. Moreover, many WRKY transcription factors in grapevines have been demonstrated to be involved in plant resistance to disease. For example, heterologous expression of *VpWRKY1*, *VpWRKY2*, *VpWRKY11*, *VqWRKY52*, *VqWRKY53*, and *VqWRKY56* enhances resistance to pathogens [32, 84, 90–92]. WRKY family members can be divided into three subfamilies [57]. WRKY33, belonging to the WRKY I family, is a pathogen-inducible transcription factor, the expression of which was shown to be essential for positively regulating resistance to *B. cinerea* [86, 93], *Alternaria brassicicola* [94], and the oomycete pathogen *Plasmopara viticola* [95]. In *A. thaliana*, AtWRKY33 induced camalexin biosynthesis after pathogen infection [86, 93, 94], and acted as an important node of the regulatory cascade [86]. In this study, a WRKY-type transcription factor, VqWRKY33, which can be induced by *E. necator*, was isolated from Danfeng-2 (Fig. 3a, Supplementary DataS5). WRKY transcription factors regulate target genes by binding to the W-box elements on target gene promoters [57]. The *VqNSTS3* promoter contained both TTGACT and TTGACC (Supplementary Data Fig. S2), which indicates that *VqNSTS3* may be directly regulated by VqWRKY33. Through Y1H, dual-luciferase, and ChIP–qPCR assays, the results showed that VqWRKY33 increased the activity of *VqNSTS3* promoter by binding directly to TTGACC on the *VqNSTS3* promoter (Fig. 3d, f–i). In this study, overexpression of *VqWRKY33* in ‘Thompson Seedless’ plants enhanced *E. necator* tolerance and increased stilbene, callose, and H_2_O_2_ accumulation, and HR cell death after inoculation, whereas RNAi plants showed the opposite phenotype (Fig. 4a–i and k). Here, compared with WT and RNAi plants, the expression of the *STS* gene in plants overexpressing *VqWRKY33* was significantly higher after inoculation with *E. necator* (Fig. 4j). Taken together, our data show that VqWRKY33 may be an important node for enhancing the expression of *VqNSTS3* in grapevine, leading to stilbenes accumulation and consequently resistance to *E. necator*.

### Phosphorylation signaling of the three-stage cascade of VqMAPK3/6-VqWRKY33 enhances *VqNSTS3* stilbene accumulation and prevents infection by pathogens

In plants, MAPK cascades can regulate plant growth processes, hormonal signaling, and the response of the plant to various stresses [96–100]. This module typically consists of three protein kinases that activate each other through phosphorylation [101]. Two MAPK cascades are known to participate in plant immunity [61, 102–106]. The MAPK cascades communicate biological signals through phosphorylation of various transcription factors [107]. Among them, WRKYs are vital substrates of MAPK cascades. For example, AtMPK3/AtMPK6-AtWRKY33 functions against *B. cinerea* and MAPK-WRKY7/8/9/11 against *Phytophthora* [83, 108]. MdMMKK4-MdMPK3-MdWRKY17 increased susceptibility to *Colletotrichum fructicola* due to SA degradation in apple [85]. However, studies of grapevine MAPK signal transduction in response to *E. necator* have not been conducted. MAPK activation is one of the earliest signaling events in plants after perception of pathogen stress [109] and participates in signal transduction of multiple defense responses [96, 110]. In *V. vinifera*, there are 14 MAPKs, 5 MAPKKs, 62 MAPKKKs, and 7 MAPKKKKs [111]. Jiao *et al.* (2017) reported that stilbene accumulation can be positively regulated by VqMAPKKK38 by mediating the activation of VqMYB14 in grapevine [59]. In this study, expression of *VqMAPKKK38*, *MEKK3*, and *MEKK5* was significantly induced after *E. necator* inoculation in Danfeng-2 (Supplementary Data Fig. S1). Then, VqMAPK3 and VqMAPK6 were activated following chitin and *E. necator* treatment (Fig. 5a and b). These results may suggest that *VqMAPKKK38*, *VqMEKK3*, and *VqMEKK5* act upstream in Danfeng-2 in transducing signal downstream of MAPK3/6 after *E. necator* inoculation. AtMPK3/MPK6 was previously reported to phosphorylate AtWRKY33 and activate camalexin biosynthesis gene expression [58]. The OsMKK4-OsMPK6 cascade plays a vital role in biosynthesis of diterpenoid phytoalexins [112]. SIPK/NTF4/WIPK-phosphorylated, WRKY33-related NbWRKY8 induced a key gene for the production of isoprenoid phytoalexins [113]. In our study, we have shown that VqMAPK3/VqMAPK6 phosphorylation of VqWRKY33 forms an accessory pathway for the regulation of stilbene biosynthesis and resistance to *E. necator* in grapevine. WRKY I family members have SP clusters, which are thought to be phosphorylated by MAPKs at the N-terminal [58, 85, 108, 113]. Four sites in the SP clusters of VqWRKY33 are important phosphorylation sites that regulate VqWRKY33-mediated *VqNSTS3* expression (Fig. 3e and h). In apple, the phosphorylation sites in the SP cluster are vital for regulating MdWRKY17-mediated *MdDMR6* activation [85]. Phosphorylation of VqWRKY33 by VqMPK3/VqMPK6 enhances its activity in promoting the expression of downstream stilbene biosynthetic genes. However, other phosphorylation sites involved in phosphorylating VqWRKY33 by VqMAPK3/6 need to be further studied. In addition, RNAi-*MAPK3/6* plants both showed increased susceptibility to *E. necator* and decreased the accumulation of stilbenes (Fig. 6). In Danfeng-2, both VqMAPK3 and VqMAPK6 showed vital functions in stilbene accumulation and *E. necator* resistance. Therefore, our study found that, under infection with *E. necator*, VqMAPK3/6 sense the stimulation of the pathogen and release phosphorylation signals, which cause downstream transcription factor VqWRKY33 to start the positive regulation of target gene *VqNSTS3*, which expresses and accumulates a large number of stilbenes, enhancing disease resistance.

### *ProVqNSTS3::VqNSTS3*-GFP moves to and wraps the haustoria to prevent pathogen invasion in transgenic *A. thaliana*

Fungal conidia invade plants by forming haustoria, which secrete proteins that degrade the host cell wall, and then invade the host plant [114]. To prevent the growth of fungi, plant cells enclose the haustoria by forming an encasement [115]. Haustorial encasements may serve as a matrix in which to concentrate plant-derived antimicrobial compounds at the plant–fungal interface, thereby poisoning the haustorium [62, 116, 117]. Several proteins that are essential for encasement formation towards PM fungus have been found in previous studies. For example, the syntaxin PEN1 (SYP121) and its closest homolog, SYP122, are required for encasement formation [62, 118]. These syntaxins are required for mediating encasement formation at the site of a fungal attack [117]. Previous studies showed that STS proteins localized to the exocarp cell wall, secondary cell wall, chloroplast, endoplasmic reticulum, and the oil bodies using immune-histochemical, immunogold electron microscopy, and laser scanning confocal microscopy techniques [48, 119, 120]. In this study, ectopic expression of *VqNSTS3* in *A. thaliana* under its own promoter showed that VqNSTS3 is crucial for postinvasive immunity against the PM pathogen by accumulating stilbenes in the transgenic lines after artificial inoculation with *G. cichoracearum* (Fig. 7f). By using laser scanning confocal microscopy, VqNSTS3 was found to localize on the plasma membrane in the absence of pathogen infection, but was actively translocated to the haustorial encasements and surrounded the haustoria when plants were challenged by *G. cichoracearum* (Fig. 7g–l). The expression enhancement of *VqNSTS3* caused large amounts of the protein VqNSTS3 to be transported to the haustorium by vesicles (Fig. 7h and i). Therefore, our results imply that the VqNSTS3-containing MVBs move to the haustorial encasements and wrap around them, preventing the growth of fungal conidia and mycelium .

**Figure 8:**
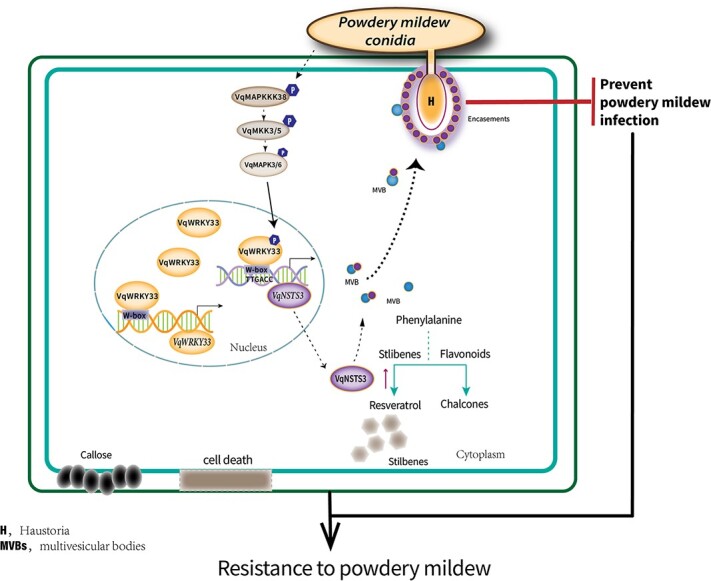
Model of the three-stage signaling cascade of VqMAPK3/6-VqWRKY33-*VqNSTS3* in Danfeng-2, enhancing stilbene accumulation and preventing infection with pathogens. *Erysiphe necator*-triggered phosphorylation of VqWRKY33 by VqMAPK3/6 enhances the binding of VqWRKY33 to the *VqNSTS3* promoter and activates *VqNSTS3* expression to promote the accumulation of stilbenes. VqWRKY33 can also activate its own expression. VqNSTS3 can be carried by MVBs that positively accumulated at haustorial encasements to inhibit the growth of PM spores.

In summary, we have discovered and identified a novel *VqNSTS3* from Danfeng-2 based on transcriptome sequencing. The novel *VqNSTS3* has the conserved domain and sequence characteristics of the *STS* family. Transgenic *VqNSTS3* plants not only rapidly produced phytoalexin but also showed HR cell death, callose accumulation, and resistance-related gene expression after *E. necator* inoculation. Grapevine VqMAPK3/6-VqWRKY33 positively regulates novel *VqNSTS3* expression and resistance to *E. necator* in grapevine immunity. It has been found that in plants that have been attacked by pathogens VqNSTS3 is actively transported to the haustorium, then surrounds the haustorium and prevents it from invading the plant (Fig. 8). These results demonstrate that the Chinese wild grapevine integrates the three-stage cascade signal to positively regulate *VqNSTS3* expression and stilbene accumulation, thereby enhancing the resistance to PM. Chinese wild grapes are valuable germplasm resources for grape disease resistance breeding.

## Materials and methods

### Plant materials

All sample tissues of Chinese wild grapevine *V. quinquangularis* accession Danfeng-2 were gathered in 2020 from the grapevine resource nursery of Northwest A & F University, Yangling, Shaanxi, China (34°20′N, 108°24′E). Callus of *V. vinifera* cultivar ‘Thompson Seedless’ was used for genetic transformation. *Arabidopsis thaliana* Columbia WT (Col-0) was cultivated and used as a transgene receptor in a growth chamber. Tobacco plants (*Nicotiana benthamiana*) were cultivated in an incubator at 22 ± 2°C with light for 16 hours.

### RNA extraction and reverse transcription–quantitative PCR

The Omega Plant RNA Kit (Omega, GA, USA) was used for RNA extraction. The FastKing RT Kit (TIANGEN, Beijing, China) was used for cDNA first-strand synthesis [92]. PerfectStart Green qPCR SuperMix (TransGen Biotech, Beijing, China) and the ABI QuantStudio 6 Flex (Applied Biosystems, Thermo Fisher, CA, USA) were used for qPCR. The 2^−ΔΔc(t)^ method was used to calculate relative expression [121]. Primers are listed in Supplementary Data Tables S1–3.

### Subcellular localization

The plasma membrane-localized marker PM-RK-mCherry [122] was co-expressed with 35S-VqMAPK3/6-GFP to validate the localization of these proteins and injected into 4-week-old tobacco leaves. And 35S-VqNSTS3-GFP construct was mobilized into the GV3101 strain of *Agrobacterium tumefaciens* and then transferred into tobacco leaves. [123]. In addition, 35S-VqWRKY33-GFP was co-expressed with 35S-AtHY5-mCherry (a marker of nucleus) into protoplasts of grapevine using the polyethylene glycol-mediated method [124]. GFP and mCherry signals were observed with confocal laser microscopy (Leica TCS SP8, Germany).

### Grapevine transformation, *E. necator* infection, histochemical staining, and microscopy

Callus isolated from ‘Thompson Seedless’ was used for grapevine transformation by *A. tumefaciens*. The transgenic transformation method was similar to the method described previously [43, 65, 123]. The third to sixth healthy and newly developed leaves from the beginning of the shoot tip were selected for *E. necator* artificial inoculation [42].

After 7 days of inoculation, leaves were gathered for trypan blue staining and calculating the number of conidiophores per colony under a microscope as described [125]. Trypan blue staining was used to visualize hyphal growth and detect cell death [27]. Callose was stained by aniline blue and visualized by UV epifluorescence. Scanning electron microscopy observation was carried out following a previously described method [15]. For transient expression experiments in grapevine, *A. tumefaciens* containing 35S-VqMAPK3/6^CA^-GFP, RNAi-*VqNSTS3,* and empty vectors was cultured in LB liquid medium, and the OD600 was adjusted to 0.6–0.7. Leaves of Danfeng-2 were vacuumed for 30 minutes by immersion in bacterial solution [126]. Then, the leaves were placed in a growth chamber for moisturizing and cultivation for 3 days and gathered for subsequent research.

### Stilbene content determination by high-performance liquid chromatography

Plant samples were freeze-dried for 48 hours, and dry weight was determined according to the volume-to-mass ratio of 1:10. Methanol was added to extract stilbene substances for 24 hours in dark. Then, the methanol extract was filtered through a 0.22-μm membrane film. High-performance liquid chromatography (HPLC) was conducted using a Nexera UHPLC LC-30A (Shimadzu, Japan). The gradient used was consistent with previous research methods [127]. Standard samples of *trans*-resveratrol (CAS: 501-36-0), piceid (CAS: 27208-80-6), piceatannol (CAS: 10083-24-6), pterostilbene (CAS: 537-42-8), and *ε*-viniferin (CAS: 62218-08-0) (Sigma–Aldrich, USA) were used to confirm retention times.

## Acknowledgements

We are grateful to Cambridge Proofreading Company for editing the text language. The research was funded by the National Natural Science Foundation of China (grant no. 32272667).

## Author contributions

Y.W. designed the research. R.L. analyzed Danfeng-2-specific novel transcripts and obtained six new stilbene synthase gene transcripts. C.Y. cloned six new *STS* genes, analyzed the gene structure and function, transiently transformed the new *STS* genes to tobacco and determined the expression of stilbenes. W.L. and C.Y. carried out the experiments, and W.L. analyzed the data. G.C. and X.W. helped with the experimental work. Y.W., C.Z., Y.X., and X.W. revised the manuscript. W.L. wrote and Y.W. reviewed and revised the manuscript.

## Data availability

All relevant data can be found within the manuscript and its supporting information.

## Conflict of interest statement

The authors declare that they have no competing interests.

## Supplementary data

Supplementary data are available at *Horticulture Research* online.

## Supplementary Material

Web_Material_uhad116
