# Supplementary material for: VqMAPK3/VqMAPK6, VqWRKY33, and VqNSTS3 constitute a regulatory node in enhancing resistance to powdery mildew in grapevine
Source: Hortic Res. 2023 May 31;10(7):uhad116. doi: 10.1093/hr/uhad116 (PMC10541564; doi:10.1093/hr/uhad116)
Supplement: Web_Material_uhad116 [file web_material_uhad116.docx]

**Supplemental Data**


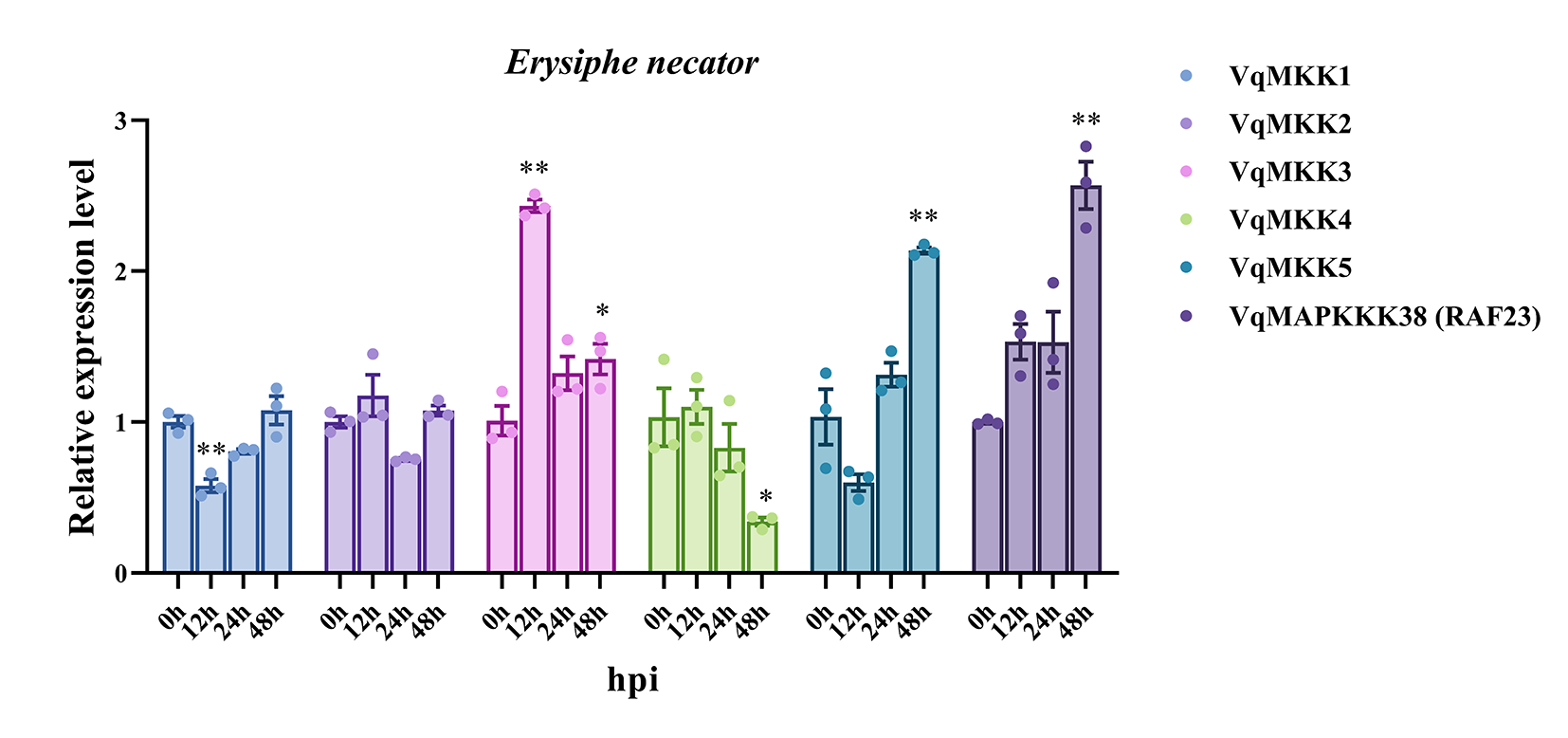


**Figure S1.** **qPCR analysis of *VqMKK1-5* and *VqMAPKKK38* from Danfeng-2 under *E. necator* inoculation**. Expression of *VqMKK1*, *VqMKK2*, *VqMKK3*, *VqMKK4*, *VqMKK5* and *VqMAPKKK38* (*RAF 23*) was detected by qPCR; hpi, hours post-inoculation. Results are shown as mean values ± SEM; n = 3. Significances were examined by one-way ANOVA, followed by Dunnett's multiple comparisons test (*, *P* < 0.05; **, *P* < 0.01).


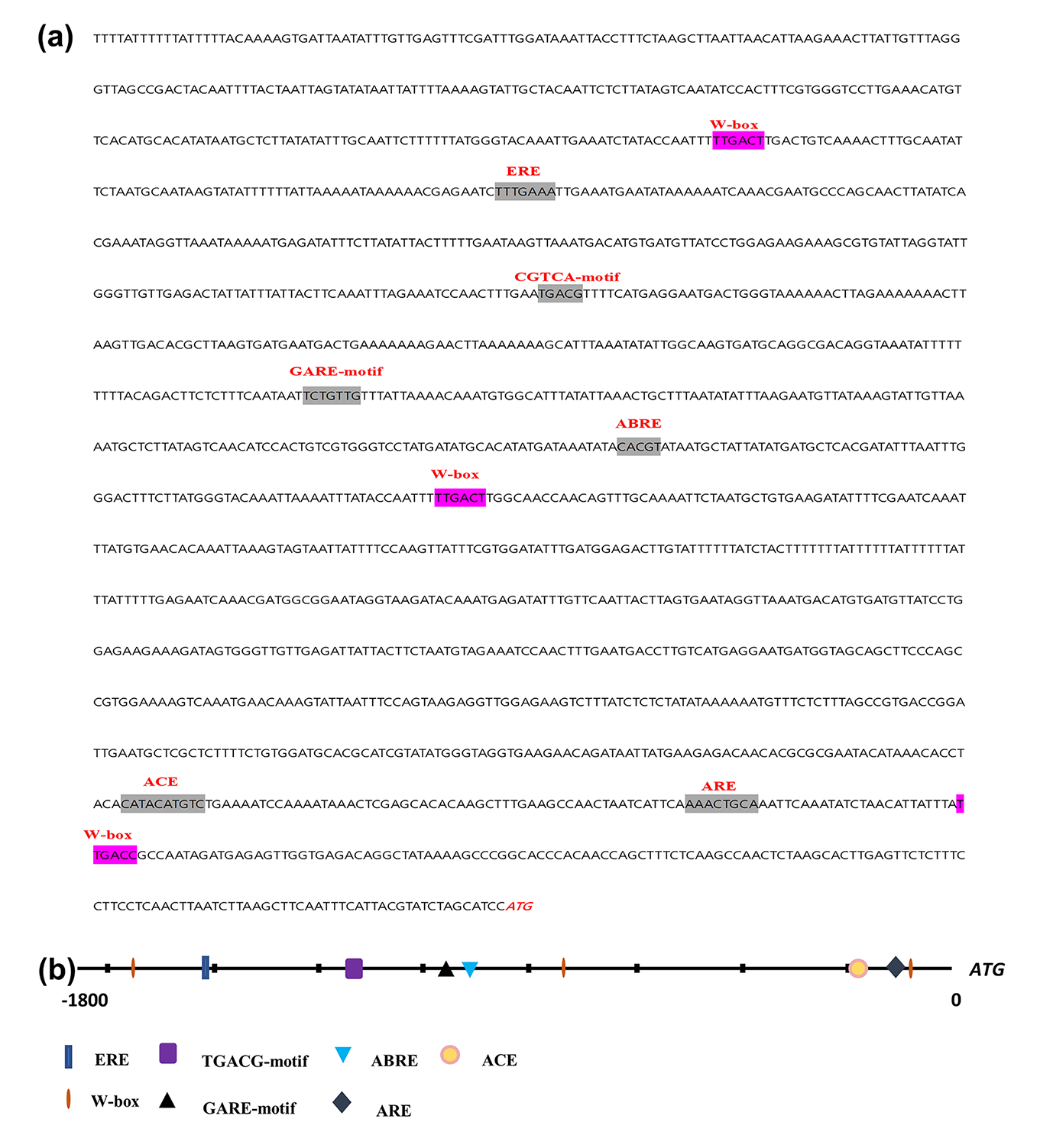


**Figure S2. Sequence analysis of *VqNSTS3* promoter elements.** **(a).** WRKY binding site W‑box is highlighted in pink. **(b).** Location of three fungal elicitor responsive and other elements found in *VqNSTS3* promoter of grapevine. Detailed information of cis-acting elements of *VqNSTS3* promoter is presented in Table S4.


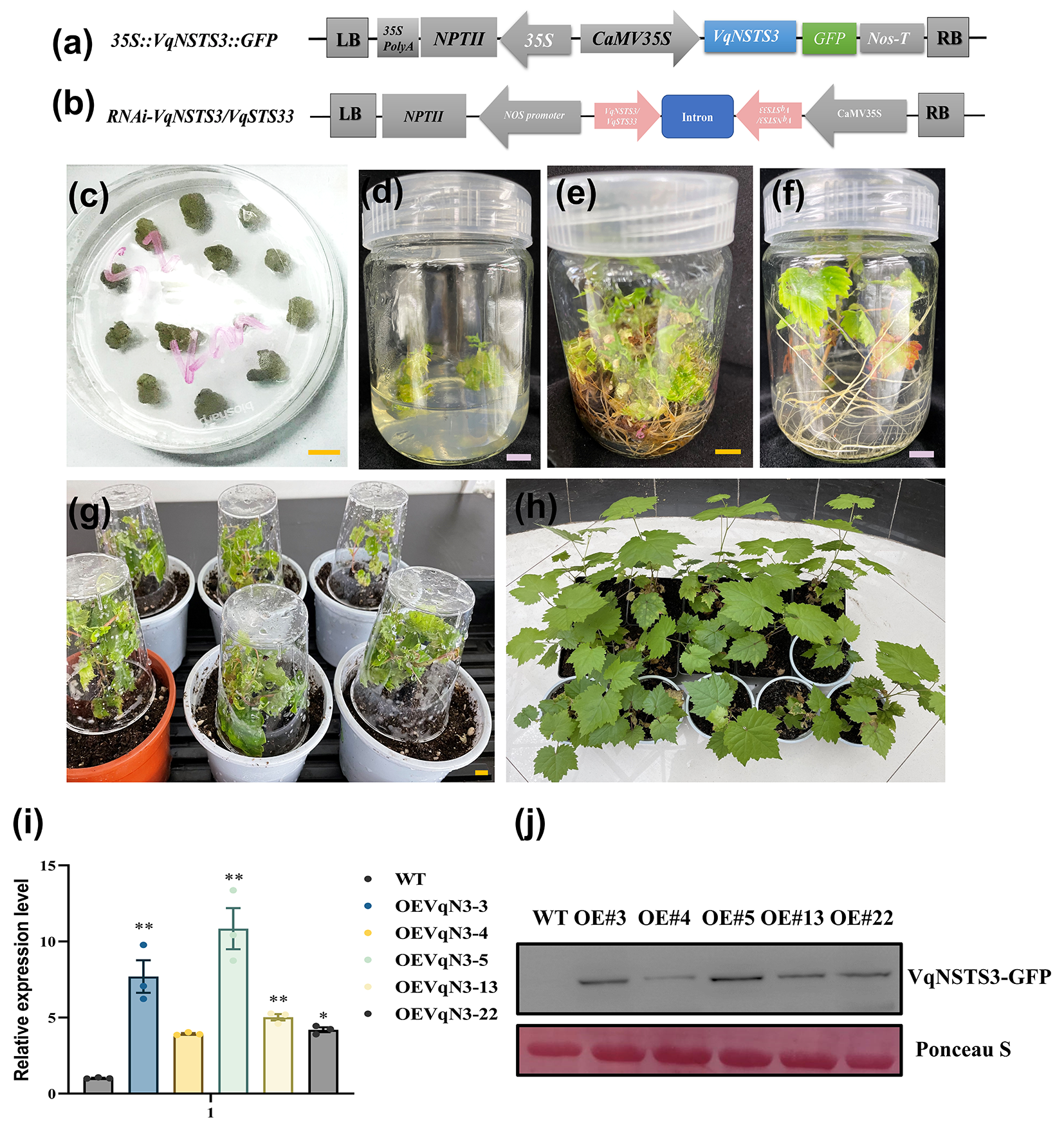


**Figure S3.** **Generation of OE*VqNSTS3* transgenic grapevine lines** **via *Agrobacterium tumefaciens* transformation.** **(a).** Diagrammatic sketch of OE*VqNSTS3* construct. **(b).** Schematic diagram of RNAi*VqNSTS3* construct. **(c-h).** Genetic transformation of *VqNSTS3* genes via *Agrobacterium tumefaciens* transformation using the callus of *Vitis vinifera* cv. ‘Thompson Seedless’ as materials. **(i).** The expression of *VqNSTS3* in transgenic and WT grapevine leaves was detected by qPCR. Results are shown as mean values ± SEM; n = 3. Significances were examined by one-way ANOVA, followed by Dunnett's multiple comparisons test (*, *P* < 0.05; **, *P* < 0.01). **(j).** Western blot detection of 35S-VqNSTS3-GFP transgenic grapevine lines.


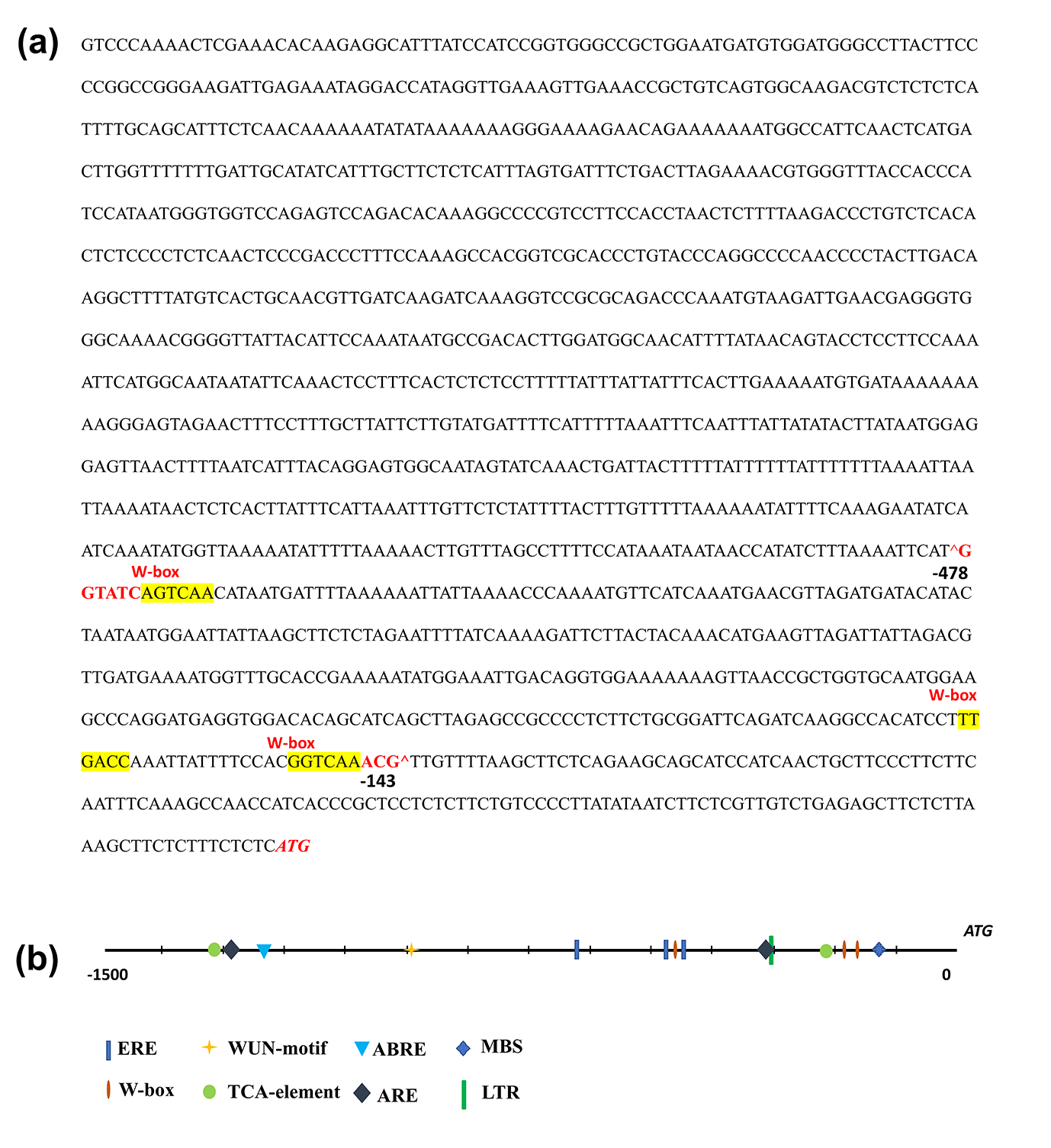


**Figure S4. Sequence analysis of *VqWRKY33* promoter elements. (a).** WRKY binding site W‑box is highlighted in yellow. **(b).** Location of cis-regulatory elements found in the *VqWRKY33* promoter of Danfeng-2. Detailed information of cis-acting elements is presented in Table S5.


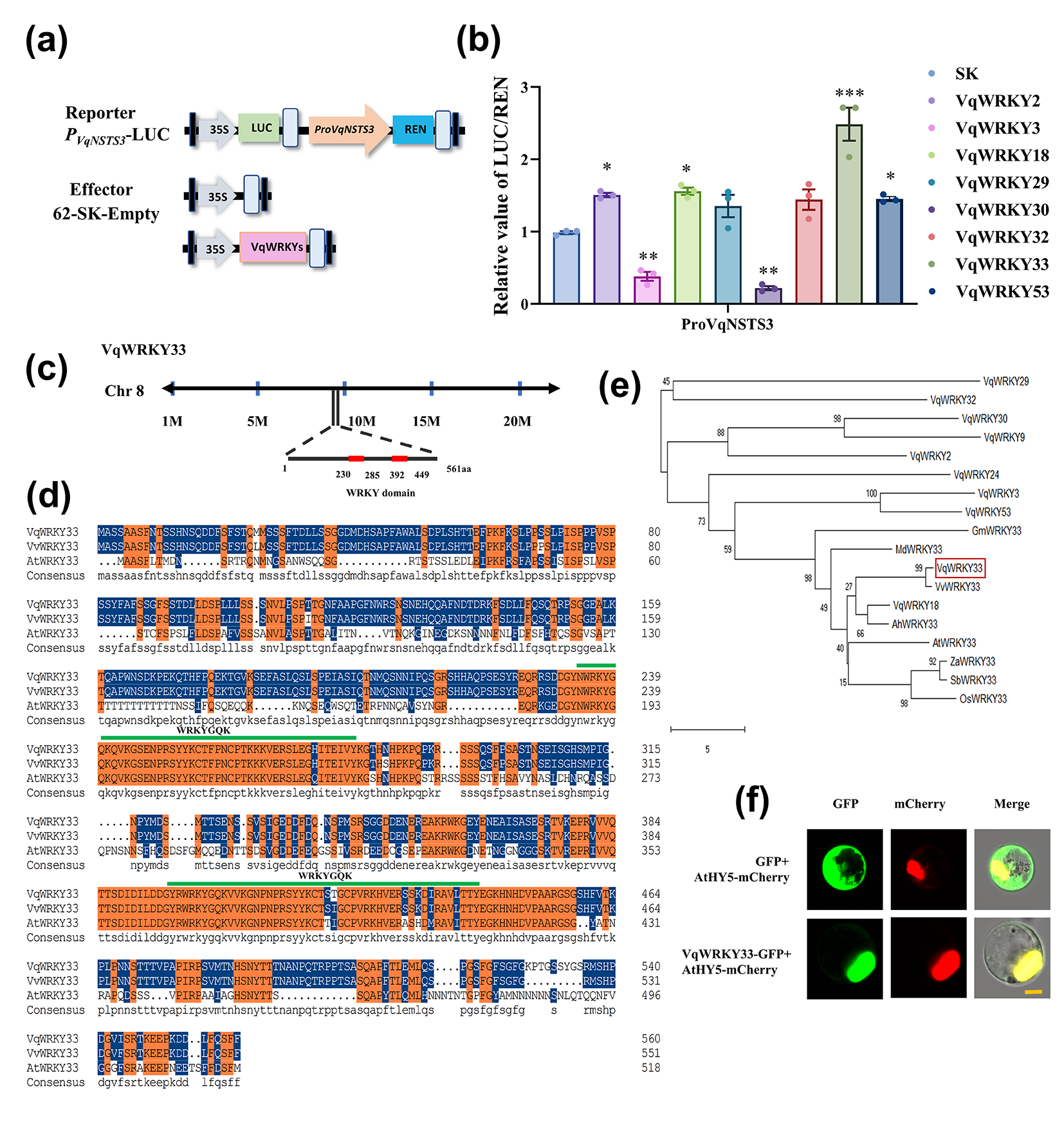


**Figure S5.** **Sequence analysis of *VqWRKY33* isolated from Danfeng-2.** **(a).** Structural constructs of dual-luciferase assays. **(b).** Ratio of luciferase activity analysis of VqWRKYs binding to *VqNSTS3* promoter. Results are shown as mean values ± SEM; n = 3. Significances were examined by one-way ANOVA, followed by Dunnett's multiple comparisons test (*, *P* < 0.05; **, *P* < 0.01; ***, *P* < 0.001). **(c).** *VqWRKY33* (VIT_08s0058g00690) located on chromosome 8. Two WRKY domains were located at VqWRKY33 from 230-285 aa and 392-449 aa. **(d).** Multiple sequence alignment between VqWRKY33, VvWRKY33 and AtWRKY33. WRKY domains (WRKYGQK) are marked with green lines. **(e).** Phylogenetic analysis of VqWRKY33 with part of WRKY transcription factors from grape and other species. **(f).** VqWRKY33 localises in the nucleus. Bars, 10 μm.


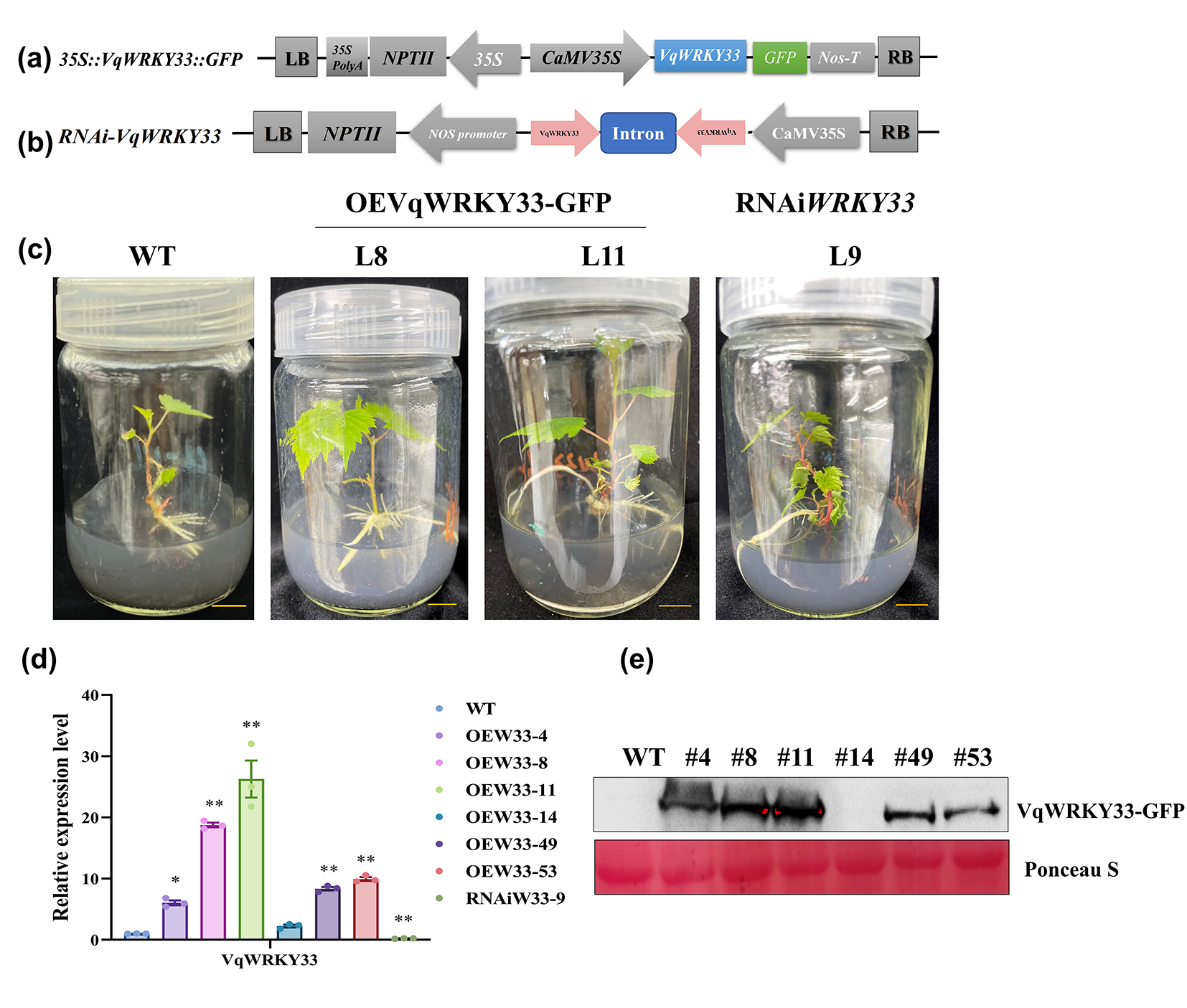


**Figure S6. Creation and identification of transgenic *VqWRKY33* grapevines.**

Diagrammatic sketch of the **(a).** OE*VqWRKY33* and **(b).** RNAi*WRKY33* construct. **(c).** Plantlets of *VqWRKY33* transgenic grapevines in tissue culture cans. **(d).** The expression of *WRKY33* in WT and transgenic grapevine leaves was detected by qPCR. Results are shown as mean values ± SEM; n = 3. Significances were examined by one-way ANOVA, followed by Dunnett's multiple comparisons test (*, *P* < 0.05; **, *P* < 0.01). **(e).** Western blot detection of 35S-VqWRKY33-GFP transgenic grapevine lines.


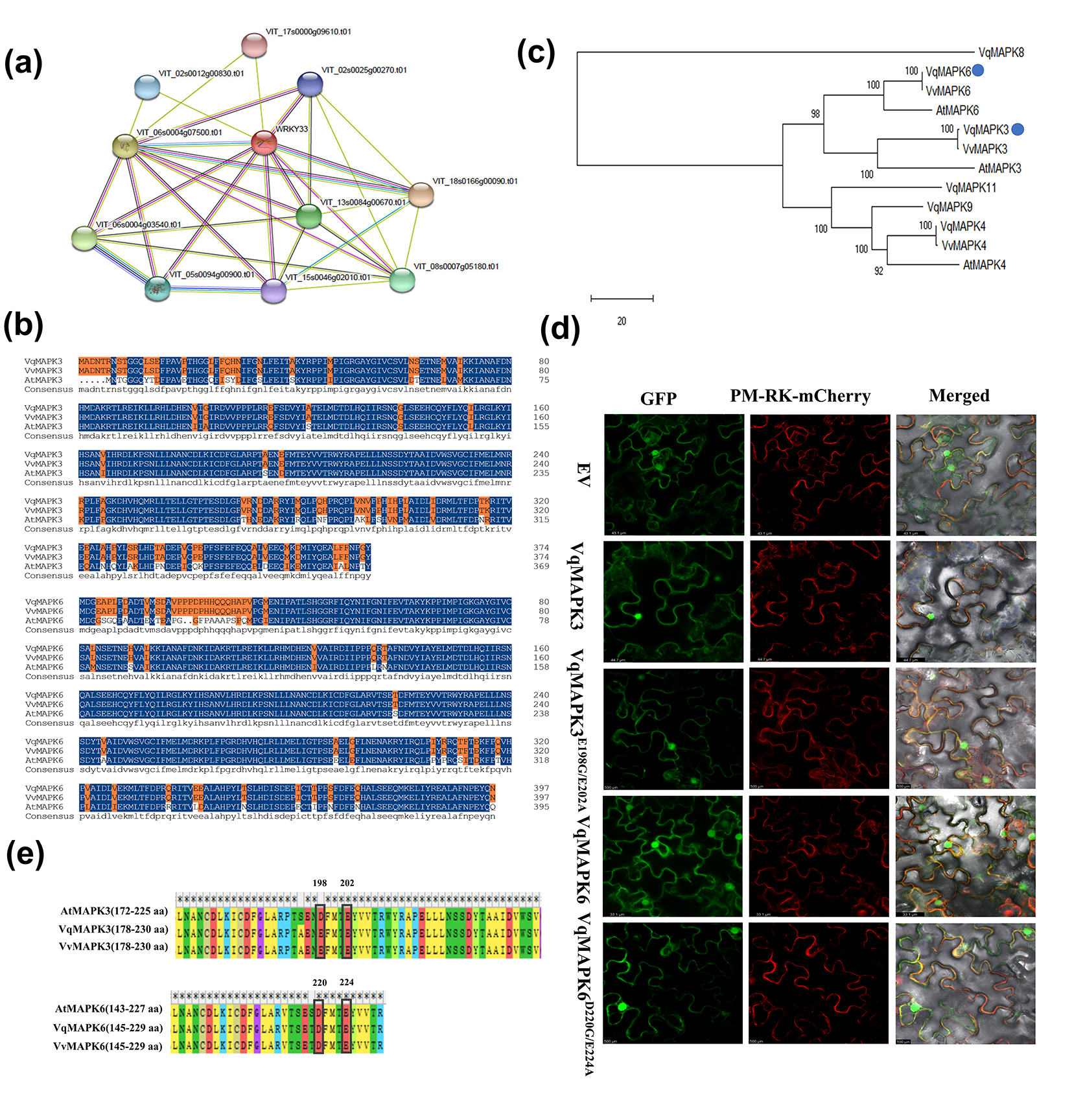


**Figure S7. Sequence analysis of *VqMAPK3* and *VqMAPK6* isolated from Danfeng-2. (a).** Co-expression network with VqWRKY33 is determined from STRING. **(b).** Multiple sequence alignment between VqMAPK3 (VIT_06s0004g03540) and VqMAPK6 (VIT_05s0094g00900). **(c).** Cluster analysis of VqMAPK3 and VqMAPK6 with part of MAPKs from grape and *Arabidopsis thaliana*. **(d).** VqMAPK3, VqMAPK6, VqMAPK3 ^E198G/E202A^ and VqMAPK6 ^D220G/E224A^ localise in the nucleus and plasma membrane. **(e).** Mutation sites of VqMAPK3 and VqMAPK6 are indicated. Black boxes represent mutation amino acids.

**
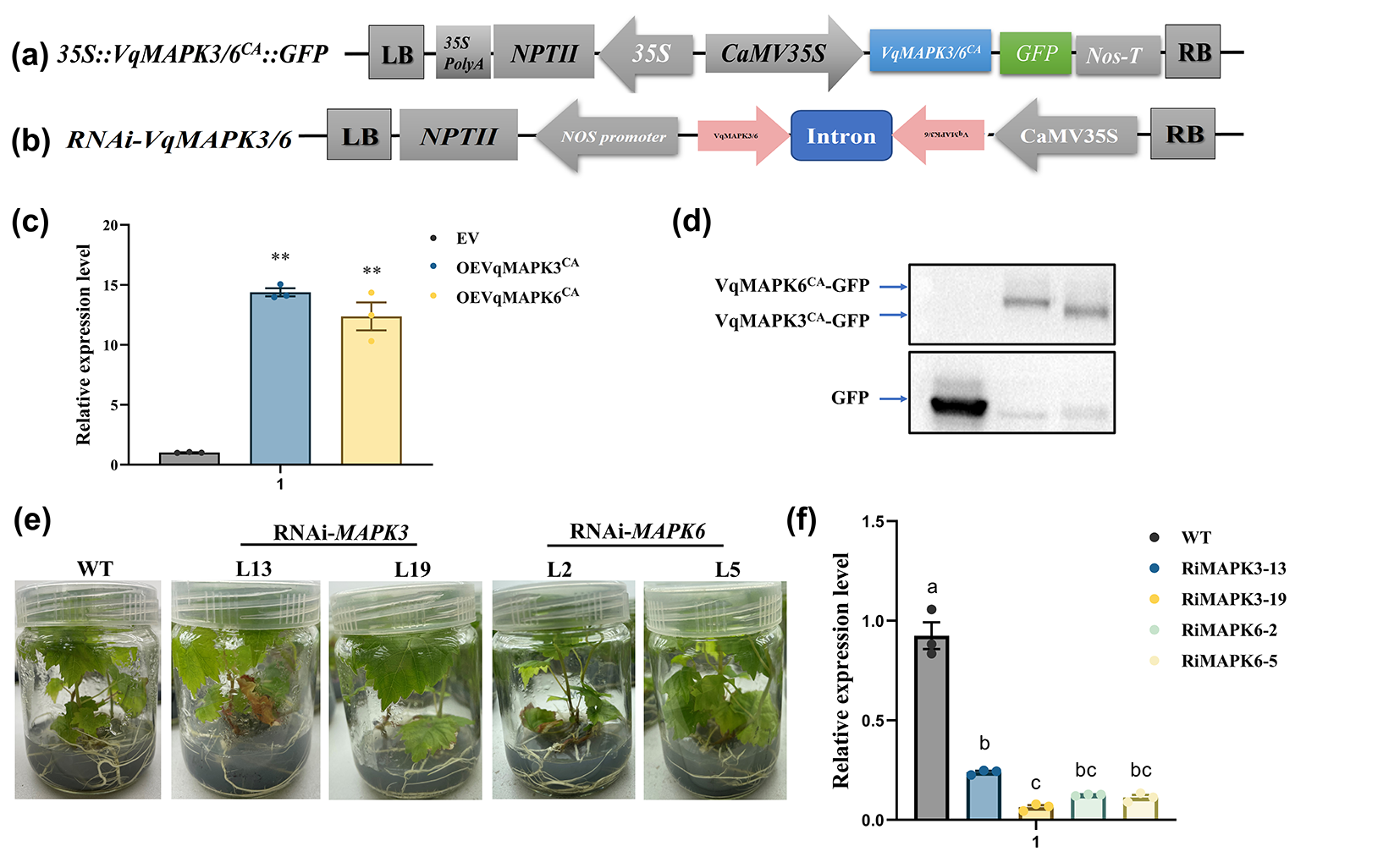
**

**Figure S8. Identification of transient expression of *VqMAPK3/6^CA^* in Danfeng-2 and transgenic RNAi*MAPK3/6* grapevines.** **(a).** Diagrammatic sketch of the OE*VqMAPK3/6^CA^* construct. **(b).** Diagrammatic sketch of the RANi-*MAPK3/6* construct. **(c).** The expression of *VqMAPK3* and *VqMAPK6* in OE*VqMAPK3^CA^*, OE*VqMAPK6^CA^* and EV leaves. Results are shown as mean values ± SEM; n = 3. Significances were examined by one-way ANOVA, followed by Dunnett's multiple comparisons test (*, *P* < 0.05; **, *P* < 0.01). **(d).** Western blot detection of 35-VqMAPK3^CA^-GFP, 35-VqMAPK6^CA^-GFP, and EV in overexpressed grapevine leaves. **(e).** Plantlets of RNAi*MAPK3/6* transgenic grapevines in tissue culture cans. **(f).** The expression of *MAPK3* and *MAPK6* in WT and RNAi plants was detected by qPCR. Results are shown as mean values ± SEM; n = 3, and different letters represent significant differences (*P < 0.05*) as determined by one-way ANOVA, followed by Tukey’s multiple comparisons test.


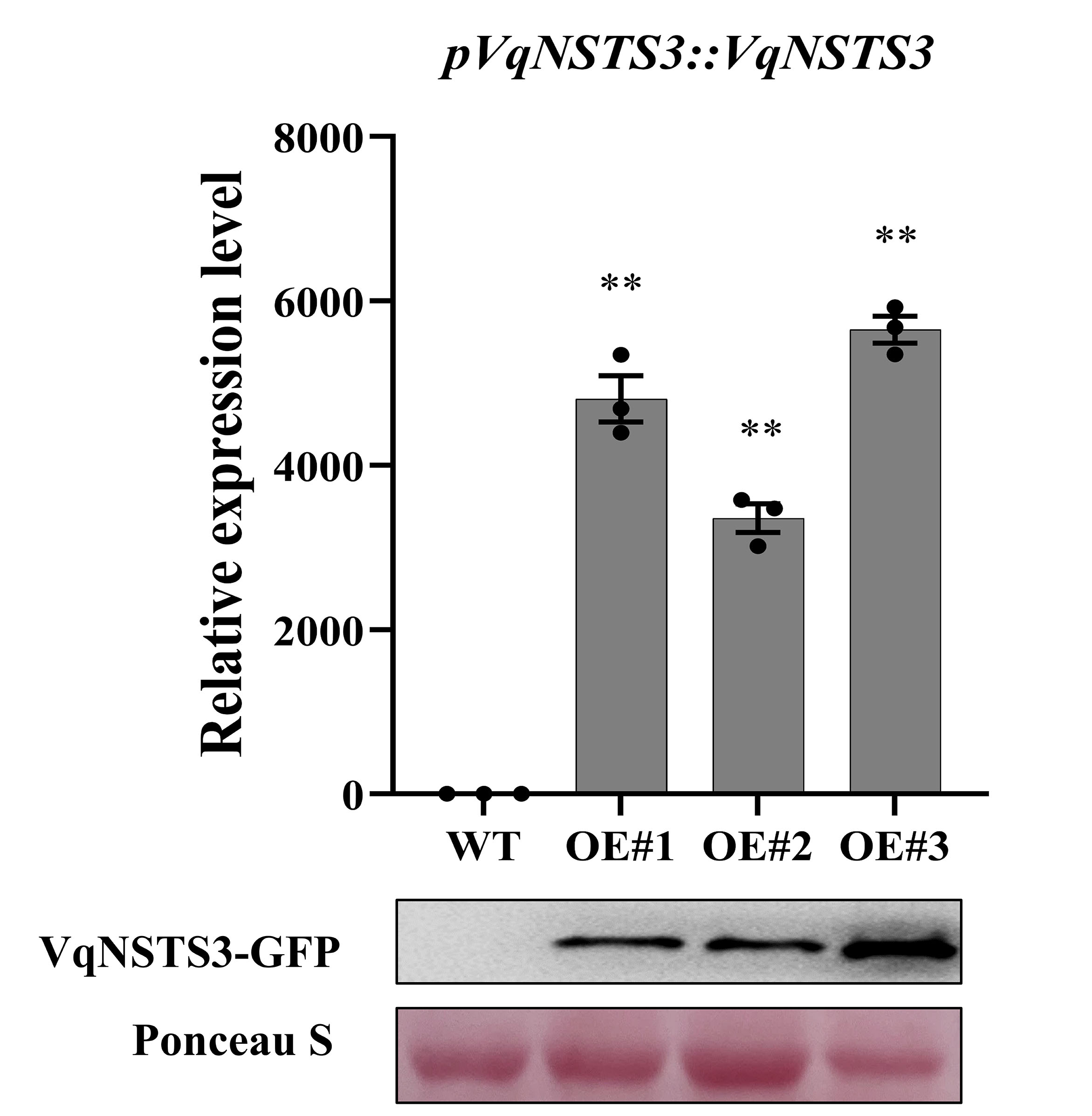


**Figure S9.** **Pro*VqNSTS3::VqNSTS3* was overexpressed in OE#1, OE#2, and OE#3 *Arabidopsis* *thaliana* transgenic lines.** Relative transcript levels of *VqNSTS3* in Col-0 and transgenic leaves was detected by qPCR. Results are shown as mean values ± SEM; n = 3. Significances were examined by one-way ANOVA, followed by Dunnett's multiple comparisons test (*, *P < 0.05*; **, *P < 0.01*). Western blot detection of Pro*VqNSTS3::VqNSTS3*-GFP transgenic *Arabidopsis* *thaliana* lines.

**Table S1** **List of primers of *VqNSTS3* used in this study**

| **Primer name** | **List of primer used in this study**  **Primers (5’-3’)** | **Use** |
| --- | --- | --- |
| VqNSTS3pro-PAbAi-F | AAT TCG AGC TCG GTA CCC GGG  AAT GCT CAG CGT GCC TAG GG | Y1H |
| VqNSTS3pro-PAbAi-R | AGC ACA TGC CTC GAG GTC GAC  CCA CAG ACG CCA TTG ATT AGT GT |  |
| VqNSTS3pro-0800LUC-F | atcgaattcctgcagcccggg CAT  CTT GAA GCC AGA AAC TTA CCC | Dual luciferase assays |
| VqNSTS3pro-0800LUC-R | cgctctagaactagtggatccCT  AATTTCCTCCACAGACGCCAT |  |
| 1-VqNSTS3/VqSTS33-frag1-BamH1-F | ctcggtacccggggatcc GGT TTA  GAT AAA CAG AAA CTC AAA GCA AC | RNAi |
| 1-VqNSTS3/VqSTS33-frag1-SpeI-R | taccttggttgcactagt GTC TCA  ATG GTT AGG CCT GGC |  |
| 2-VqNSTS3/VqSTS33-frag1-BglII-F | gtcgactctagcagatct GGT TTA  GAT AAA CAG AAA CTC AAA GCA AC |  |
| 2-VqNSTS3/VqSTS33-frag1-Xbal1-R | aaccatctgcagtctagaGTC TCA  ATG GTT AGG CCT GGC |  |
| VqNSTS3Pro-GUS-F | gaccatgattacgccaagctt CCA  AGC TGG TAG TGG GAT CAC | GUS activity assays |
| VqNSTS3Pro-GUS-R | ggactgaccacccggggatcc  CAT GGA TGC TAG ATA CGT AAT GAA ATT G |  |
| VqNSTS3-CHIPqPCR-F S1 | TAT ACC AAT TTT TGA CTT  GAC TGT CAA AAC TTT GCA | CHIP-qPCR |
| VqNSTS3-CHIPqPCR-R S1 | TGG GCA TTC GTT TGA TTT TTT  ATA TTC ATT TCA ATT TCA AAG |  |
| VqNSTS3-CHIPqPCR-F S2 | CCT ATG ATA TGC ACA TAT  GAT AAA TAT ACA CGT ATA ATG C |  |
| VqNSTS3-CHIPqPCR-R S2 | GGT TGC CAA GTC AAA AAT  TGG TAT AAA TTT TAA TTT GTA C |  |
| VqNSTS3-CHIPqPCR-F S3 | CTA ACA TTA TTT ATT GAC  CGC CAA TAG ATG AGA G |  |
| VqNSTS3-CHIPqPCR-R S3 | TGA GGA AGG AAA GAG AAC  TCA AGT GCT TAG |  |
| RT-VqNSTS3/VqSTS33-F | GGTTTAGATAAACAGAAACTCAAAGCAAC | RT-qPCR |
| RT-VqNSTS3/VqSTS33-R | GTC TCA ATG GTT AGG CCT GGC |  |
| RT-VqSTSs-F | CAAGCCCTTTTTGGTGATG |  |
| RT-VqSTSs-R | CCACAAGTGAAAGGTGAGTCC |  |
| RT-VqCHIT4C-F | gcaaccgatgttgacatatca |  |
| RT-VqCHIT4C-R | cgtcgccctagcaagtgag |  |
| RT-VqRBOHD-F | caccaccatgcttcagtccctccat |  |
| RT-VqRBOHD-R | agcgatcttcttgaagacttgtcgcc |  |
| RT-VqPR1-F | GTGTAGGAGTCCATTAGC |  |
| RT-VqPR1-R | GCATTGAGGTAGTCTTGT |  |
| RT-VqPR5-F | TGATTCAGGTTCGGTGGTTGC |  |
| RT-VqPR5-R | ATGTCTGGGGGGTGCTGTGGT |  |
| RT-VqGAPDH-F | TTCTCGTTGAGGGCTATTCCA |  |
| RT-VqGAPDH-R | CCACAGACTTCATCGGTGACA |  |

**Table S2** **List of primers of *VqWRKYs* used in this study**

| **Primer name** | **List of primer used in this study**  **Primers (5’-3’)** | **Use** |
| --- | --- | --- |
| VqWRKY33-AD-F | GGC CAG TGA ATT CCA CCC GGG  ATG GAT CAC AGT GCT CCA TTC GC | Y1H |
| VqWRKY33-AD-R | CAG CTC GAG CTC GAT GGA TCC TCA GCA  AAA AAA TGA CTG GAA CAA GTC ATC |  |
| VqW33pro-pAbAi-F | AAT TCG AGC TCG GTA CCC GGG ATC TAC  GGA ATT CAA TAT CAA TCA TTA CCT C |  |
| VqW33pro-pAbAi-R | AGC ACA TGC CTC GAG GTC GAC GCA  GAG GAA GCC ATG AGA GAA AG |  |
| VqWRKY33-1307-F | GTA TCT AGA ACT AGT GGA TCC  ATG GCT TCC TCT GCT GCT AGT TT | In-plant expression |
| VqWRKY33-1307-R | GGG CCC CCC CTC GAG GTC GAC GCA  AAA AAA TGA CTG GAA CAA GTC ATC |  |
| VqWRKY33-2300-F | GAG CTC GGT ACC CGG GGA TCC  ATG GCT TCC TCT GCT GCT AGT TT |  |
| VqWRKY33-2300-R | CTT GCT CAC CAT GGT GTC GAC GCA  AAA AAA TGA CTG GAA CAA GTC ATC |  |
| VqWRKY33-NE-F | agtggatccgtcgacctcgagATG  GCT TCC TCT GCT GCT AGT TT | BiFC |
| VqWRKY33-NE-R | ctcctacccgggagcggtaccGCA  AAA AAA TGA CTG GAA CAA GTC ATC |  |
| VqWRKY33-LUCn_F BamHI | GAGCTCGGTACCCGGGGATCCAAAA  ATG GCT TCC TCT GCT GCT AGT TT | Split-luc assays |
| VqWRKY33-LUCn_R XBaI | ACCTCCTGAACCTCCTCTAGA  GCA AAA AAA TGA CTG GAA CAA GTC ATC |  |
| VqWRKY33pro-0800LUC-F | atcgaattcctgcagcccggg  GTC CCA AAA CTC GAA ACA CAA GAG | Dual luciferase assays |
| VqWRKY33pro-0800LUC-R | cgctctagaactagtggatccAGA  GGA AGC CAT GAG AGA AAG AG |  |
| VqWRKY2-SK-F | cgctctagaactagtggatcc  ATGGAGGCTGCTGCTTTGGAG |  |
| VqWRKY2-SK-R | atcgaattcctgcagcccgggTCA  CTGGATATTATCTTTGTGATCAGAAG |  |
| VqWRKY3-SK-F | cgctctagaactagtggatcc  ATGGATAGCTTCTCCACTCTCTTTC |  |
| VqWRKY3-SK-R | atcgaattcctgcagcccgggTCA TCATTAAAAGGAAGCATAAACTTGCATC |  |
| VqWRKY18-SK-F | cgctctagaactagtggatccATG  GGT TCT TCC TCT GGG AGC |  |
| VqWRKY18-SK-R | atcgaattcctgcagcccgggTCA  ACA GAG CAG AGA CTC GAA TAA C |  |
| VqWRKY29-SK-F | cgctctagaactagtggatccATG  GAC GGC AGA TTC AAC AGT AAC |  |
| VqWRKY29-SK-R | atcgaattcctgcagcccgggTCA  GCT TGT GGT CCC ACA CAT G |  |
| VqWRKY30-SK-F | cgctctagaactagtggatccATG  GCT ATG GAT AGT TCT AAT TGG ATG |  |
| VqWRKY30-SK-R | atcgaattcctgcagcccgggTCA  CCA TTT TTC AGT TTG ATT ATG GTG G |  |
| VqWRKY32-SK-F | cgctctagaactagtggatcc  ATGGATGAACTAATGGCTGCCTG |  |
| VqWRKY32-SK-R | atcgaattcctgcagcccgggTCA  CCT AAT GCT ATA GCG TTT ACT AAT ATC |  |
| VqWRKY33-SK-F | cgctctagaactagtggatccATG  GCT TCC TCT GCT GCT AGT TT |  |
| VqWRKY33-SK-R | atcgaattcctgcagcccgggTCA  GCA AAA AAA TGA CTG GAA CAA GTC |  |
| VqWRKY53-SK-F | cgctctagaactagtggatccATGG  ATGGAAGGCCACCAAATACTTTTTC |  |
| VqWRKY53 SK-R | atcgaattcctgcagcccgggTCA TTATGAGATGGGAAAATATGATTGC |  |
| 1-VqWRKY33-frag1-BamH1-F | ctcggtacccggggatcc CTCTCCGATCCTTTAAGTCATACAAC | RNAi |
| 1-VqWRKY33-frag1-SpeI-R | taccttggttgcactagtCAG  CAA AAT TCC CAG TGG TTG GA |  |
| 2-VqWRKY33-frag1-BglII-F | gtcgactctagcagatct CTCTCCGATCCTTTAAGTCATACAAC |  |
| 2-VqWRKY33-Xbal1-R | aaccatctgcagtctagaCAG  CAA AAT TCC CAG TGG TTG GA |  |
| VqWRKY33Pro-489-GUS-F | gaccatgattacgccaagctt  TTT AAA ATT CAT GGT ATC GGT CAA CAT AAT G | GUS activity assays |
| VqWRKY33Pro-GUS-R | ggactgaccacccggggatcc  GAG AGA AAG AGA AGC TTT AAG AGA AGC |  |
| VqWRKY33Pro-147-GUS-F | gaccatgattacgccaagctt  ACG TTG TTT TAA GCT TCT CAG AAG CAG |  |
| VqWRKY33-CHIPqPCR-F | TCA GCT TAG AGC CGC CCC TC | CHIP-qPCR |
| VqWRKY33-CHIPqPCR-R | GAA GCA GTT GAT GGA TGC TGC TTC |  |
| RT-VqWRKY33-F | CCTCCCGTCTCTCCTTCTTC | RT-qPCR |
| RT-VqWRKY33-R | TCATTGAAAGCCTGCTGGTG |  |

**Table S3** **List of primers of *VqMAPK3* and *VqMAPK6* used in this study**

| **Primer name** | **List of primer used in this study**  **Primers (5’-3’)** | **Use** |
| --- | --- | --- |
| VqMAPK3-1307-F | GTA TCT AGA ACT AGT GGA TCC  ATG GCT GAC AAT ACG AGG AAC TC | In-plant expression |
| VqMAPK3-1307-R | GGG CCC CCC CTC GAG GTC GAC  AGC GTA ACC TGG ATT GAA AAA TAA AGC |  |
| VqMAPK3-2300-F | GAG CTC GGT ACC CGG GGA TCC  ATG GCT GAC AAT ACG AGG AAC TC |  |
| VqMAPK3-2300-R | CTT GCT CAC CAT GGT GTC GAC  AGC GTA ACC TGG ATT GAA AAA TAA AGC |  |
| VqMAPK6-1307-F | GTA TCT AGA ACT AGT GGA TCC  ATG GAT GGA GAA GCG CCA CTG |  |
| VqMAPK6-1307-R | GGG CCC CCC CTC GAG GTC GAC  ATA GTT CTG ATA CTC GGG GTT AAA TGC |  |
| VqMAPK6-2300-F | GAG CTC GGT ACC CGG GGA TCC  ATG GAT GGA GAA GCG CCA CTG |  |
| VqMAPK6-2300-R | CTT GCT CAC CAT GGT GTC GAC  ATA GTT CTG ATA CTC GGG GTT AAA TGC |  |
| VqMAPK3-CE-F | gggactctagaggatctcgag  ATG GCT GAC AAT ACG AGG AAC TC | BiFC |
| VqMAPK3-CE-R | atcgtatgggtacatggtacc  AGC GTA ACC TGG ATT GAA AAA TAA AGC |  |
| VqMAPK6-CE-F | gggactctagaggatctcgag  ATG GAT GGA GAA GCG CCA CTG |  |
| VqMAPK6-CE-R | atcgtatgggtacatggtacc  ATA GTT CTG ATA CTC GGG GTT AAA TGC |  |
| VqMAPK3-LUCc_F BamHI | TGGATCAGGTGGAGCGGATCCAAAA  ATG GCT GAC AAT ACG AGG AAC TC | Split-luc assays |
| VqMAPK3-LUCc_R XBaI | GTCGACTCTAGTTTATCT  AGA AGC GTA ACC TGG ATT GAA AAA TAA AGC |  |
| VqMAPK6-LUCc_F BamHI | TGGATCAGGTGGAGCGGATCCAAAA  ATG GAT GGA GAA GCG CCA CTG |  |
| VqMAPK6-LUCc_R XBaI | GTCGACTCTAGTTTATCT  AGA ATA GTT CTG ATA CTC GGG GTT AAA TGC |  |
| VqMAPK3-E198G-F | CAGAGAATGgGTTCATGACAGAAT  ATGTTGTTACGAGA | Overlap PCR for mutation of VqMAPKs |
| VqMAPK3-E198G-R | CATGAACcCATTCTCTGCAGTTGGACGAGCAA |  |
| VqMAPK3-E198G/E202A-F | GGGTTCATGACAGcATATGTTGTT  ACGAGATGGTACAGGG |  |
| VqMAPK3-E198G/E202A-R | TATgCTGTCATGAACCCATTCTCTGCAGTTGG |  |
| VqMAPK6-D220F-F | CCTCTGAAACTGgTTTCATGACAG  AATATGTTGTTACGAG |  |
| VqMAPK6-D220F-R | GAAAcCAGTTTCAGAGGTGACACGAGCTAGCC |  |
| VqMAPK6-D220F/E224A-F | CTGGTTTCATGACAGcATATGTT  GTTACGAGATGGTACCGA |  |
| VqMAPK6-D220F/E224A-R | TgCTGTCATGAAACCAGTTTCAGAGGTGACAC |  |
| 1-VqMAPK3-BamH1-F | ctcggtacccggggatcc  ATG GAT GGA GAA GCG CCA CTG | RNAi |
| 1-VqMAPK3-SpeI-R | taccttggttgcact  agt CTG GAT GAA TCT GCC GCC G |  |
| 2-VqMAPK3-BglII-F | gtcgactctagcagatct  ATG GAT GGA GAA GCG CCA CTG |  |
| 2-VqMAPK3-Xbal1-R | aaccatctgcagtctagaCTG  GAT GAA TCT GCC GCC G |  |
| 1-VqMAPK6-BamH1-F | ctcggtacccggggatcc  ATG GCT GAC AAT ACG AGG AAC TC |  |
| 1-VqMAPK6-SpeI-R | taccttggttgcactagt ACC GAT CGG CAT GAT CGG AG |  |
| 2-VqMAPK6-BglII-F | gtcgactctagcagatct  ATG GCT GAC AAT ACG AGG AAC TC |  |
| 2-VqMAPK6-Xbal1-R | aaccatctgcagtctaga  ACC GAT CGG CAT GAT CGG AG |  |

**Table S4** **Details of cis-acting elements involved in pathogen- and stress-responsive expression of *VqNSTS3* promoter**

| Cis-element | Number of cis-element | Function |
| --- | --- | --- |
| W-box* | 3 | Fungal elicitor responsive element |
| TGACG-motif | 1 | MeJA-responsiveness |
| GARE-motif | 1 | Gibberellin-responsiveness |
| ERE | 1 | Ethylene-responsiveness |
| ARE | 1 | Anaerobic-responsive |
| ABRE | 1 | ABA-responsiveness |

* **Note**：*VqNSTS3* promoter carries three specific fungal elicitor responsive elements

**Table S5** **Details of cis-acting elements involved in pathogen- and stress-responsive expression of *VqWRKY33* promoter**

| Cis-element | Number of cis-element | Function |
| --- | --- | --- |
| W-box* | 3 | Fungal elicitor responsive element |
| TCA-element* | 3 | SA-responsiveness |
| ABRE | 1 | ABA-responsiveness |
| LTR | 1 | low-temperature responsiveness |
| MBS | 1 | involved in drought-inducibility |
| ARE | 2 | Anaerobic-responsive |
| WUN-motif | 1 | wound-responsive element |
| ERE | 3 | Ethylene-responsiveness |

* Note: *VqWRKY33* promoter carries three specific fungal elicitor response elements and three salicylic acid response elements.

**Table S6 List of the VqWRKY33-interacting proteins.**

| VIT_18s0166g00090 | Uncharacterized protein |
| --- | --- |
| VIT_06s0004g07500 | Wrky transcription factor wrky24; Uncharacterized protein |
| VIT_06s0004g03540 | Mitogen-activated protein kinase 3; Belongs to the protein kinase superfamily. Ser/Thr protein kinase family. MAP kinase subfamily |
| VIT_13s0084g00670 | Uncharacterized protein |
| VIT_08s0007g05180 | Sigma factor binding protein 2, chloroplastic; Uncharacterized protein |
| VIT_05s0094g00900 | Mitogen-activated protein kinase homolog mmk1; Belongs to the protein kinase superfamily. Ser/Thr protein kinase family. MAP kinase subfamily |
| VIT_02s0012g00830 | Uncharacterized protein; Belongs to the expansin family |
| VIT_02s0025g00270 | Mitogen-activated protein kinase homolog mmk2; Belongs to the protein kinase superfamily. Ser/Thr protein kinase family. MAP kinase subfamily |
| VIT_15s0046g02010 | Mitogen-activated protein kinase homolog mmk2; Belongs to the protein kinase superfamily. Ser/Thr protein kinase family. MAP kinase subfamily |
| VIT_17s0000g09610 | Cytochrome p450 family 71 subfamily a; Uncharacterized protein; Belongs to the cytochrome P450 family |

**Table S7 Stilbenes contents of OE*VqNSTS3* and RNAi*VqNSTS3* plants under artificial inoculation with *Erysiphe necato*r**

| **Stilbenes contents (μg/g) Mean±SEM** | | | | | |
| --- | --- | --- | --- | --- | --- |
|  | *trans*-resveratrol | piceid | pterostilbene | *ε*-viniferin | piceatannol |
| WT-7dpi  (Thompson Seedless) | Nd | 56.0±0.30 | Nd | Nd | 35.27±10.27 |
| OE*VqNSTS3*-7dpi  (Thompson Seedless) | 72.95±14.41 | 733.3±149.70 | 17.91±3.29 | 89.79±16.43 | 219.6±45.00 |
| EV-5dpi  (Danfeng-2) | 61.43±6.83 | 156.6±13.87 | 25.56±2.66 | 48.88±6.65 | 75.86±5.63 |
| RNAi*VqNSTS3*-5dpi  (Danfeng-2) | 16.43±3.57 | 66.7±1.20 | 14.95±0.53 | 28.72±0.74 | 10.64±1.36 |

**Table S8 Stilbenes contents of OE*VqWRKY33* and RNAi*WRKY33* plants under artificial inoculation with *Erysiphe necato*r**

| **Stilbenes contents (μg/g) Mean±SEM** | | | | | |
| --- | --- | --- | --- | --- | --- |
|  | *trans*-resveratrol | piceid | pterostilbene | *ε*-viniferin | piceatannol |
| WT-7dpi  (Thompson Seedless) | Nd | 49.33±3.49 | Nd | Nd | 35.27±5.32 |
| OE*VqWRKY33*-7dpi  (Thompson Seedless) | 34.37±0.80 | 332.4±42.28 | 13.98±4.56 | 41.71±2.37 | 81.66±6.05 |
| RNAi*WRKY33*-7dpi (Thompson Seedless) | Nd | 842.7±46.43 | Nd | Nd | Nd |

**Table S9 Stilbenes contents of OE*VqMAPK3/6^CA^* and RNAi*MAPK3/6* plants under artificial inoculation with *Erysiphe necato*r**

| **Stilbenes contents (μg/g) Mean±SEM** | | | | |
| --- | --- | --- | --- | --- |
|  | *trans*-resveratrol | piceid | *ε*-viniferin | piceatannol |
| EV-5dpi  (Danfeng-2) | 69.02±7.67 | 175.9±15.58 | 24.20±4.18 | 85.22±6.33 |
| OE*VqMAPK3^CA^*-5dpi  (Danfeng-2) | 162.6±35.27 | 659.9±11.83 | 135.7±4.51 | 111.6±3.55 |
| OE*VqMAPK6^CA^*-5dpi  (Danfeng-2) | 132.4±2.18 | 831.7±29.70 | 139.3±3.64 | 136.6±1.60 |
| WT-7dpi  (Thompson Seedless) | Nd | 854.2±147.0 | Nd | 36.52±0.79 |
| RNAi*MAPK3*-7dpi  (Thompson Seedless) | Nd | 50.2±15.2 | Nd | Nd |
| RNAi*MAPK6*-7dpi  (Thompson Seedless) | Nd | 72.3±3.9 | Nd | Nd |

**Table S10 Stilbenes contents of Pro*VqNSTS3::VqNSTS3* transgenic *Arabidopsis* *thaliana* under artificial inoculation with *Golovinomyces cichoracearum***

| **Stilbenes contents (μg/g) Mean±SEM** | | |
| --- | --- | --- |
|  | *trans*-resveratrol | piceid |
| Col-0-7dpi  (*Arabidopsis thaliana*) | Nd | Nd |
| Pro*VqNSTS3::VqNSTS3*-7dpi  (OE#1)  (*Arabidopsis thaliana*) | 44.76±5.53 | 66.13±8.80 |
| Pro*VqNSTS3::VqNSTS3*-7dpi  (OE#2)  (*Arabidopsis thaliana*) | 47.07±3.40 | 63.72±7.45 |
| Pro*VqNSTS3::VqNSTS3*-7dpi  (OE#3)  (*Arabidopsis thaliana*) | 49.34±2.27 | 69.03±4.94 |

**Supplemental Methods**

***Arabidopsis* *thaliana* transformation for the identification resistance to pathogen and confocal laser scanning microscopy**

The *Agrobacterium* strain GV3101 containing the Pro*VqNSTS3::*VqNSTS3-GFP was grown in a liquid Luria broth medium and the OD600 was adjusted to 0.5-0.6. Resuspended bacterial liquid was used to infect the flowers of *Arabidopsis thaliana* ^1^. Four-week-old leaves of T3 Pro*VqNSTS3::*VqNSTS3-GFP transgenic *Arabidopsis thaliana* plants were inoculated with *Golovinomyces cichoracearum* cultivated on *pad4* mutants ^2^. Leaves were harvested in a Calcofluor white solution for observing spores and mycelium. The method for staining membrane lipids with FM4-64 and the use of confocal laser scanning microscopy were executed following a previously described method ^3^. Images were captured using a Leica TCS SP8 SR confocal laser-scanning microscope.

**GUS activity assays**

The promoters of *VqWRKY33* or *VqNSTS3* from Danfeng-2 were separately inserted into the pC0390-GUS vector, and transferred into *Agrobacterium tumefaciens* GV3101 strain and transiently infiltrated in *N. benthamiana* or Danfeng-2 leaves. After 72 h of cultivation, GUS activity and staining were carried out as described previously^4^. 35S-GUS was used as a positive control.

**Dual‑luciferase assays**

Dual-luciferase assays were performed according to the previously described method ^5,6^. The CDSs of *VqWRKYs* were inserted into the pGreenII 62-SK vector and the promoters of *VqNSTS3* and *VqWRKY33* were separately inserted into the pGreenII 0800-LUC vector. *Agrobacterium tumefaciens* GV3101 carrying the corresponding constructs were resuspended in an MES buffer injected into 4-week-old tobacco leaves. The ratio of enzyme activities of Firefy luciferase (LUC) and Renilla luciferase (REN) was carried out by Dual Luciferase Reporter Gene Assay Kit (Beyotime, Beijng, China).

**Yeast one-hybrid assays**

Matchmaker™ Gold Yeast One-Hybrid System (Clontech, Palo Alto, USA) was adopted for experimental validation. The promoters of *VqNSTS3* and *VqWRKY33* were inserted into the pAbAi vector as baits. Next, *BstbI* or *BbsI* linearized pAbAi vectors and then integrated into the Y1HGold strain. The CDSs of *VqWRKYs* were inserted into pGADT7 and transfected into baits separately. The empty AD vector was transfected into baits as negative control. Transformants were grown on medium with SD/−Leu with AbA (150ng/ml).

**ChIP-qPCR assays**

Three grams of Danfeng-2 leaves overexpressing VqWRKY33-GFP were taken, cross-linked with 0.5% formaldehyde under vacuum for 10 min, added with 2M glycine, and vacuumed for 5 min to terminate cross-linking. Then the ChIP assay was carried out following the EpiQuik Plant ChIP Kit (EpigenTek, Brooklyn, NY, USA). Anti-GFP (TransGen Biotech) was added for immunoprecipitation. Mouse normal IgG was used as a negative control. Subsequently, immunoprecipitated and input DNA were quantified by qPCR. Primers used for qPCR are listed in Supplementary Table S1.

**Split-luciferase complementation assays**

The CDS of *VqWRKY33* was inserted into pCAMBIA1300-nLUC, and the CDSs of *VqMAPK3* and *VqMAPK6* were cloned into pCAMBIA1300-cLUC. Different constructs were transiently infiltrated in *N. benthamiana* and luciferase activity was examined as described ^7^. A CCD imaging system (Lumazone Pylon 2048B) was used for observing luciferase complementation.

**Bimolecular fluorescence complementation assays (BiFC)**

BiFC assays were carried out following the method described^8^. The CDS of *VqWRKY33* was inserted into the pSPYNE vector. The CDSs of *VqMAPK3* and *VqMAPK6* were inserted into pSPYCE vector. Different constructs were transiently infiltrated in *N. benthamiana*. The YFP signals were observed with confocal laser microscopy (Leica TCS SP8, Germany).

**Co-immunoprecipitation assays**

CDSs of *VqMAPK3* and *VqMAPK6* were introduced into the pCAMBIA2300-GFP vector. Meanwhile, the CDS of *VqWRKY33* was introduced into Myc-pCAMBIA1307. *Agrobacterium tumefaciens* GV3101 carrying different combinations of plasmids injected in *N. benthamiana* leaves. Proteins were extracted from 1ml of Cell lysis buffer for Western and IP (Beyotime, Shanghai, China). Myc antibody (TransGen Biotech) was added to protein extract and incubated overnight at 4°C, then 50 μL of Protein G Magnetic Beads (no.70024, Cell Signaling Technology, Inc) were added for another 40 min to capture the target complex. The magnetic beads were collected with a magnetic grate and washed five times with a cold NP40 lysis buffer (Beyotime, Shanghai, China). Proteins were collected from the magnetic beads adding 5 × loading buffer and boiled for 5 min for use in immunoblot analyses. Phos-tag gel (F4002, APExBio) was used to detect the phosphorylation of VqWRKY33.

**Supplemental References**

1. Clough, S.J. & Bent, A.F. Floral dip: a simplified method for *Agrobacterium*-mediated transformation of *Arabidopsis thaliana*. *the Plant Journal* **16**, 735-743 (1998).

2. Xiao, S., Ellwood, S., Findlay, K., Oliver, R.P. & Turner, J.G. Characterization of three loci controlling resistance of Arabidopsis thaliana accession Ms-0 to two powdery mildew diseases. *the Plant Journal* **12**, 757-768 (1997).

3. Meyer, D., Pajonk, S., Micali, C., O'Connell, R. & Schulze-Lefert, P. Extracellular transport and integration of plant secretory proteins into pathogen-induced cell wall compartments. *the Plant Journal* **57**, 986-999 (2009).

4. Xu, W., Yu, Y., Ding, J., Hua, Z. & Wang, Y. Characterization of a novel stilbene synthase promoter involved in pathogen- and stress-inducible expression from Chinese wild *Vitis pseudoreticulata*. *Planta* **231**, 475-487 (2010).

5. Wang, D., Jiang, C., Li, R. & Wang, Y. VqbZIP1 isolated from Chinese wild *Vitis quinquangularis* is involved in the ABA signaling pathway and regulates stilbene synthesis. *Plant Science* **287**, 110202 (2019).

6. Yu, Y. *et al.* The Chinese wild grapevine (*Vitis pseudoreticulata*) E3 ubiquitin ligase *Erysiphe necator*-induced RING finger protein 1 (EIRP1) activates plant defense responses by inducing proteolysis of the VpWRKY11 transcription factor. *New Phytologist* **200**, 834-846 (2013).

7. Vielba-Fernández, A. *et al.* Fungicide resistance in powdery mildew fungi. *Microorganisms* **8**, 1431 (2020).

8. Wang, D., Jiang, C., Liu, W. & Wang, Y. The WRKY53 transcription factor enhances stilbene synthesis and disease resistance by interacting with MYB14 and MYB15 in Chinese wild grape. *Journal of Experimental Botany* **71**, 3211-3226 (2020).
